# Leprosy: treatment, prevention, immune response and gene function

**DOI:** 10.3389/fimmu.2024.1298749

**Published:** 2024-02-19

**Authors:** Xiang Li, Yun Ma, Guoli Li, Guangjie Jin, Li Xu, Yunhui Li, Pingmin Wei, Lianhua Zhang

**Affiliations:** ^1^ Department of Epidemiology and Health Statistics, School of Public Health, Southeast University, Nanjing, China; ^2^ Chronic Infectious Disease Control Section, Nantong Center for Disease Control and Prevention, Nantong, China; ^3^ Department of Chronic Infectious Disease Control and Prevention, Jiangsu Provincial Center for Disease Control and Prevention, Nanjing, China

**Keywords:** leprosy, MDT, prophylaxis, rifapentine, immune cells, susceptibility gene

## Abstract

Since the leprosy cases have fallen dramatically, the incidence of leprosy has remained stable over the past years, indicating that multidrug therapy seems unable to eradicate leprosy. More seriously, the emergence of rifampicin-resistant strains also affects the effectiveness of treatment. Immunoprophylaxis was mainly carried out through vaccination with the BCG but also included vaccines such as LepVax and MiP. Meanwhile, it is well known that the infection and pathogenesis largely depend on the host’s genetic background and immunity, with the onset of the disease being genetically regulated. The immune process heavily influences the clinical course of the disease. However, the impact of immune processes and genetic regulation of leprosy on pathogenesis and immunological levels is largely unknown. Therefore, we summarize the latest research progress in leprosy treatment, prevention, immunity and gene function. The comprehensive research in these areas will help elucidate the pathogenesis of leprosy and provide a basis for developing leprosy elimination strategies.

## 1 Introduction

It has been reported that the risk of leprosy infection is associated with factors such as spatial distance between patients, genetics, disease type and age. The prevention of leprosy has become a hot issue in recent years, especially among people chronically exposed to Mycobacterium leprae (M.leprae), such as household close contacts (HHC) (1). Experts point out that early diagnosis, reduction of infection rates and cutting off transmission routes contribute to disease control. Therefore, the World Health Organisation (WHO) proposed early detection of cases and screening of high-risk populations as key strategic objectives in the “Global Leprosy Strategy 2016-2020” (2).

The patients display a wide range of clinical manifestations due to the distinct immune responses specific to each individual. Ridley-Jopling classification system classifies patients into five categories: Tuberculoid (TT), Lepromatous (LL), Borderline Lepromatous (BL), Borderline Borderline (BB), and Borderline Tuberculoid (BT). The TT type exhibits few skin lesions, well-developed granulomas, and a lower bacterial load. On the other hand, the LL type shows abundant bacteria in the affected areas but fewer lymphocytes and granuloma formation. Most patients belonged to intermediate phenotypes, including BL, BB, and BT (3). Additionally, the WHO classifies patients into Multibacillary (MB) and Paucibacillary (PB) types based on skin patch results, where those with more than five skin lesions are considered MB, and those with fewer lesions as PB (4). Therefore, leprosy was considered an ideal disease to study the immune interrelationship between the pathogen and the host at the time of infection. However, the pathogenesis of leprosy remains obscure due to the lack of an ideal animal model that accurately replicates vital features of leprosy observed in humans.

The disease has long been recognized as a familial aggregation disease, with host genetics being the main factor influencing pathogenesis. With technological advances, genome-wide linkage and analyses have provided evidence of genetic function. Comprehensive analyses of susceptibility genes revealed a complex network of interactions between related genes, and more than 30 susceptibility genes were known to be associated with leprosy (5, 6). In addition, there is a shared genetic background between leprosy and certain inflammatory and autoimmune diseases such as Parkinson’s (PD) and Crohn’s (CD) (7, 8).

## 2 Drug regimen and preventive measures

### 2.1 Transition from monotherapy with dapsone to multidrug therapy

**Figure 1:**
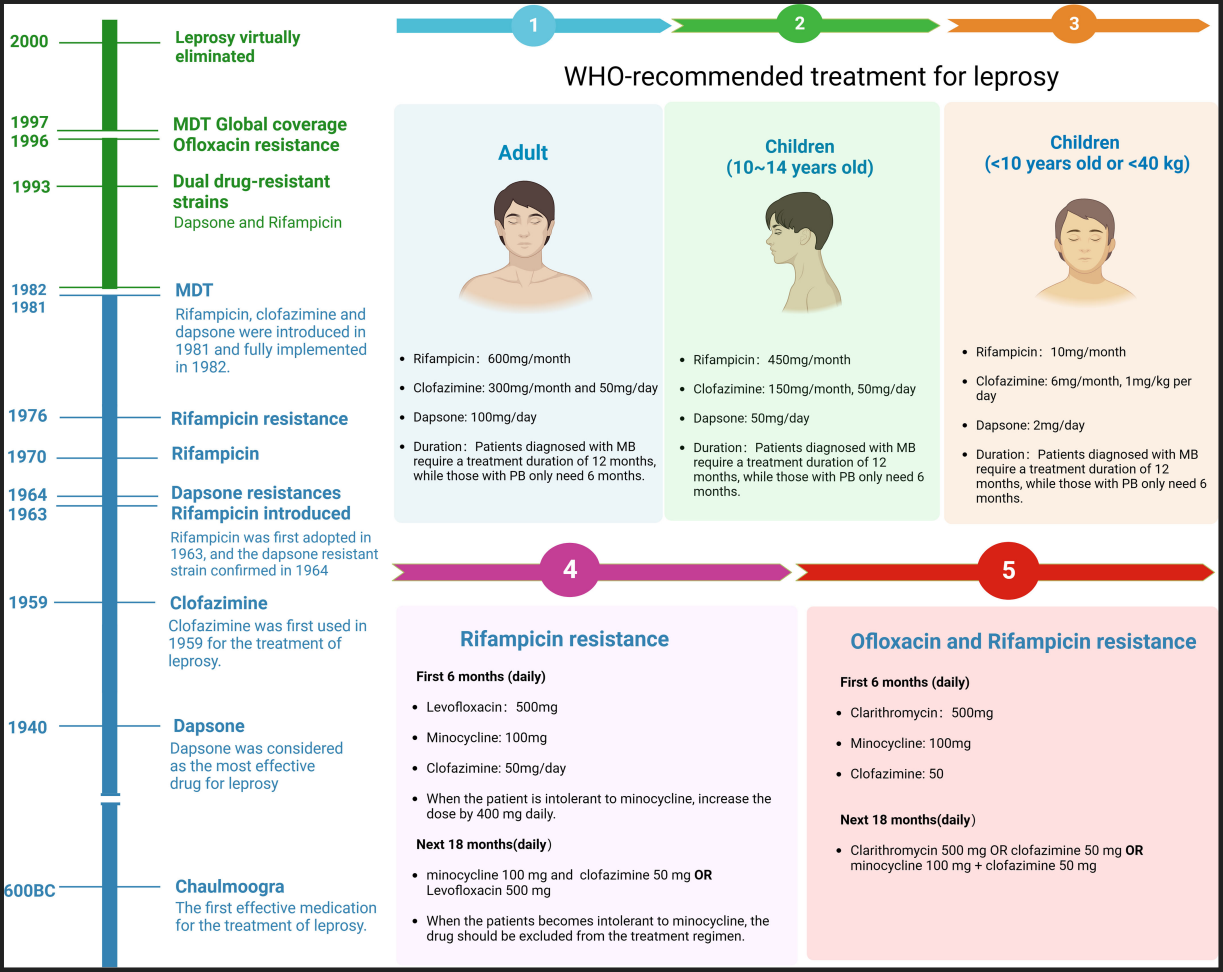
History of leprosy treatment and MDT protocol. The chart shows that leprosy was largely eliminated till 2000, and the first dapsone-resistant strain was confirmed in 1963. The right side of the chart shows the WHO’s guidelines for adults and children and the protocols following Rifampicin or Ofloxacin resistance.

Chaulmoogra oil was first used in leprosy, but its effectiveness was controversial (9). Since 1940, dapsone has been considered the most effective antibiotic in treatment. However, the patients required lifelong medication, and the prevalence remained high. In 1964, Shepard et al. identified the first dapsone-resistant strain using the mouse footpad model. Consequently, the WHO recommended MDT for leprosy treatment in 1981. The therapy has made leprosy less of a significant public health problem. The disability rate had fallen dramatically, and millions of patients were no longer disabled. It is worth noting that dapsone resistance had already exceeded 20% before MDT introduction (10). See more details in **Figure 1**.

At the beginning of the 21st century, the World Health Organization (WHO) evaluated two leprosy treatment regimens: single-dose ROM (Rifampicin, Ofloxacin, and minocycline) and conventional regimens (Rifampicin, Clofazimine, and Dapsone). The ROM regimen was found to be the best option for treating patients with PB, but it was not suitable for patients with MB or leprosy with erythema nodosum leprosum (ENL) (11). As a result, the WHO explicitly recommended treating all types of patients with the conventional regimen. The introduction of this program has successfully eliminated leprosy as a significant public health problem. However, despite the positive outcomes observed in clinical trials, the primary criticisms and concerns surrounding this harmonized regimen include the unnecessary inclusion of clofazimine in PB patients and the associated risk of relapse in MB patients. In summary, leprosy treatment faces three main challenges. Firstly, the disease persists to varying degrees in numerous countries and regions globally, with limited access to quality healthcare, particularly in economically disadvantaged areas. Next, there still needs to be an understanding of the pathogenesis and immune interaction mechanisms contributing to the continued spread of the bacteria. Finally, resistance to the core drug rifampicin has emerged, potentially compromising the effectiveness of multidrug therapy (MDT). Given the low prevalence of leprosy, focusing on chemoprophylaxis and immunoprophylaxis should be central to its control efforts.

### 2.2 Epidemiological impact of different treatment options

Before clarifying the epidemiological impact of different treatment options, it is crucial to emphasise two essential indicators: the number of persons treated in a given period, which can be seen as an approximation of the prevalence rate. Second, the number of new cases detected annually in a specific period can be seen as an approximation of the incidence rate. Shortening the treatment duration was the pivotal factor in achieving the elimination target set by WHO (12). After MDT was introduced, the treatment duration for MB patients was reduced from 24 to 12 months from 1990 to 1998. Reduced treatment time has been a vital element in the efforts to eliminate leprosy. In 1977, more than twelve million patients were under treatment. However, these patients require treatment for up to 4-10 years, while others require lifelong treatment. The global count of patients receiving treatment decreased from 5 to 3 million until 1991 (13).

After the endorsement for the widespread adoption of MDT, it took approximately 15 years to achieve global coverage for all registered leprosy patients. The number of individuals undergoing treatment further declined to approximately 600,000 by 2000. After WHO declared the goal of eradicating the disease, the number of newly detected cases dropped to 260,000 nationwide in 2007 and has remained stable ever since. Experts attributed this rapid decline in case detection to a reduction in infection transmission resulting from a decrease in the community load of M. leprae (14). It is important to note that various factors beyond drug treatment influence the spread of leprosy. Factors such as access to clean water, improved education, and close contact between family members and patients also play significant roles. In recent years, the detection rate of new cases has decreased significantly: the global number of leprosy cases in 2020 was 127,396, a decrease of 27.7% compared to 2019 (202,185 cases). However, the latest report from the WHO in 2023 stated that there were 174,087 new cases in 2022, a further 36.6% increase from 2020. New cases are concentrated in Southeast Asia, with three countries - Brazil, India and Indonesia - accounting for 78.1% (15). It is worth noting that many countries still do not have adequate disease notification services, which can lead to underreporting.

### 2.3 Emergence of drug resistance during MDT application

The molecular mechanisms of drug resistance in leprosy remain unclear. Horizontal gene transfer has been ruled out as irrelevant to drug resistance, and no novel genetic elements have been identified in the M. leprae genome (16). The gold standard for detecting drug resistance involves assessing clinical symptoms and conducting PCR tests to identify mutations in drug resistance-determining (DRDR) regions. Dihydropteroate synthase, RNA polymerase, and DNA gyrase, encoded by the Folp1, RpoB, and GyrA genes, are known drug targets for dapsone, rifampicin, and ofloxacin. However, there was limited knowledge of the resistance targets to Clarithromycin, minocycline and clofazimine. It was known that the drug target of Clarithromycin is 23SrRNA (10, 17–19). Drug-target interactions are controlled by atomic bonds between amino acid residues. Mutations can severely affect these interactions, leading to changes in protein stability and reducing the affinity between target proteins and ligands, ultimately causing drug resistance. Based on these insights, studies are systematically screening potent compounds from large libraries against newly hypothesized disease-causing protein structures for the development of novel anti-leprosy drugs or vaccines, such as the MabA and LipU proteins (20, 21).

Disease surveillance was necessary in endemic regions all the time till its eradication. In 2008, the WHO International Leprosy Elimination Program established a multi-country surveillance network. The first monitoring results revealed that out of 1,932 patients, 154 cases showed drug-resistant strains (8%), with resistance rates of 3.8% for dapsone, 4.5% for clofazimine, and 1.10% for ofloxacin. Multi-drug Resistance (MDR) was observed in 1.24% of cases in 19 countries that participated in the sentinel surveillance network, with resistant strains reported in six endemic countries (India, Brazil, Japan, Indonesia, Philippines, Colombia and China). Among them, Brazil (32 cases), India(18 cases), and Colombia(9 cases) have reported the most Rifampicin-resistant strains, and it has been observed that tranexamic acid and rifampicin resistance are prevalent among relapsed patients (22). In addition, relapsing patients are more likely to develop resistance to dapsone and rifampicin. These findings indicate that, except for specific highly endemic areas, drug resistance rates are relatively low in most regions. Ofloxacin is the preferred drug for treating drug-resistant cases, and it exhibits relatively low resistance rates. However, in some rural areas, the inappropriate use of the drug to treat tuberculosis has increased drug resistance (23). Clinically, bacteria still maintain a relatively high sensitivity to clofazimine, which could be attributed to several factors (24). In conclusion, current efforts to combat drug resistance should focus on careful post-treatment follow-up of treated individuals, rapid identification of strains that may develop secondary resistance, and more resistance surveys in endemic areas, focusing on relapse cases.

### 2.4 Chemoprophylaxis strategies for leprosy

Dapsone and acedapsone were the first chemical drugs used for leprosy prophylaxis. It was reported that Acedapsone is given weekly for 2-3 years, while dapsone is administered every 10 weeks for 7 months (25, 26). Initial trials of acedapsone were conducted in school-age patients, with subsequent findings showing protection ability in close contact (27, 28). However, the results of a meta-analysis showed that dapsone was protective for household contacts, but the number of treatments needed to prevent new cases was also higher (29). Meanwhile, the protection rate of acedapsone was only 56.7% (30). Simultaneously, the enthusiasm for chemoprophylaxis diminished following the introduction of short-term MDT and the initiation of the leprosy elimination strategy. Consequently, these two drugs were withdrawn from the programme. In 1988, single-dose rifampicin (SDR) was introduced to address drug resistance and other concerns for leprosy chemoprophylaxis (31–33). The Bangladesh Randomised Controlled Trial (COLEP) demonstrated an overall efficacy of around 60% within the first two years of SDR. However, the level of protection varied based on exposure, with up to 70% protection observed for unrelated individuals and only about 25% for blood relatives (parents or children) (34, 35). The limited effect of chemoprophylaxis might be attributed to earlier infection with M.leprae or the drug’s shorter half-life. Nevertheless, SDR as a post-exposure prophylaxis (PEP) for contacts of leprosy-affected individuals can reduce the risk of developing leprosy by up to 60% (36, 37). In 2018, SDR-PEP was formally included in the WHO Guidelines for diagnosing, treating and preventing Leprosy (38). It has proven highly cost-effective when integrated into routine leprosy control with established contact tracing.

In recent years, a groundbreaking study by Wang et al. involved 7450 household contacts divided into three groups: rifapentine, rifampicin, and control groups. The study found that the cumulative incidence rate in the rifapentine group was 92% lower than that in the control group for household contacts with more than four years of exposure (39). The effect of single-dose rifapentine surpassed SDR. Consequently, single-dose rifapentine and SDR were included in China’s national leprosy elimination program. Despite the success of chemoprophylaxis, it cannot provide lasting protection, and long-term dependence may lead to the development of drug resistance. As a result, future efforts will focus on developing durable immune responses induced by vaccines.

### 2.5 Immunoprophylactic strategies against leprosy

An effective vaccine should induce solid and long-lasting cellular immunity to bacterial antigens. The Bacillus Calmette-Guérin (BCG) vaccine, primarily used to prevent neonatal tuberculosis, can potentially protect against leprosy. As a result, the WHO recommends maintaining BCG vaccination in all high-burden leprosy areas (40). In Brazil, the Ministry of Health initially recommended routine BCG vaccination for household contacts of newly diagnosed patients, and the practice was later extended to include two doses. However, the available evidence does not support revaccination, as studies have found a high likelihood of PB patients after revaccination (37, 41). The mechanism may involve the activation of specific T cells and the overexpression of innate immune cells. The protective effect of BCG in childhood is around 60% (37, 41). Lwin et al. concluded that BCG alone is unlikely to be the ultimate solution for leprosy control (42). Schuring et al. demonstrated an 80% protective effect of SDR when the exposed person had received the BCG vaccine as part of a childhood vaccination program, compared to 58% without BCG vaccination (43). In the MALTALEP trial, SDR after BCG vaccination reduced the incidence of PB leprosy in exposed persons by 42%. At the same time, this reduction was not statistically significant due to the limited number of cases following SDR vaccination. In addition, it remains difficult to determine the extent to which SDR suppressed cases of leprosy following BCG vaccination, as many of the cases arose prior to the SDR intervention. Consequently, it is still recommended that contacts of new cases of leprosy should be investigated and then SDR should be carried out. Additionally, potential vaccine candidates have been considered (Mip/LepVax) (44–46). MIP is a whole-cell vaccine modified from BCG, while LepVax is a multivalent recombinant protein vaccine that safely induces T-cell responses. It has been suggested that LepVax may be preferred as a therapeutic vaccine rather than for prophylactic immunization. In summary, immunoprophylactic strategies have shown promise future in combating leprosy. However, further research is needed to develop more effective long-term solutions.

## 3 Nerve damage and immune response in leprosy

### 3.1 Leprosy reaction

Pathological immune reactions, i.e., Reversal Reaction (RR), Erythema Nodosum (ENL), occur in about 30% or more leprosy patients. Among them, RR is the most common cause of nerve injury, appearing at the beginning of treatment or up to 2 months before its initiation. It is accompanied by generalized skin lesions and polar neuroinflammation (47). ENL patients are characterized by the sudden onset of generalized erythema and painful nodules, primarily in patients with BL/LL type, and thalidomide is often recommended for treating strategy (48). Immune complex deposition and complement cascade reactions formed by ENL could also cause hypersensitivity or vasculitis (49). In recent years, the Lucio phenomenon is known as diffuse leprosy and is characterized by extensive violet plaques and maculopapular infiltrates, but this group of patients is relatively rare (50). As for Lucio’s Phenomenon, even though some articles cite it as a form of leprosy reaction (Type 3 Reaction, T3R), but it remains controversial whether it is a specific type of leprosy reaction. In addition, Covid-19 or smallpox vaccination may cause leprosy reactions in patients (51, 52).

### 3.2 The function of innate immune cells in the pathogenesis of leprosy

#### 3.2.1 Dendritic cells

Skin tissue is the first line of defense against M.leprae infection and DCs play an essential role in immune activation. Langerhans cells (LCs), a specific type of DCs in the epidermis, closely interact with keratinocytes and mediate antiviral activity by presenting antigens through CD1a and langerin protein (CD207). LCs have a higher antigen presentation efficiency than other DCs. The mechanism is described by Hunger et al (53, 54). LCs may capture specific antigens by expressing CD207, and at least some of these antigens can be presented to CD1a-restricted T cells for clone. Notably, CD1a antigens were also involved in the presentation of lipids and glycolipids, and they were more expressed in RR patients. LCs may induce autophagic response through IFN-γ co-localization, promoting the differentiation of CD8 T cells into cytotoxic T cells for bactericidal effects. While LCs have been studied more extensively than dermal DCs, granulomas and skin lesions are predominantly associated with dermal DCs (55). Identifying whether CD207+ cells in the diseased dermis are migrating LCs from the epidermis or another type of resident dermal DCs remains challenging. CD123 and FXIIIa are biomarkers for plasma cell-like DCs (Pd) and dermal DCs (Dd), respectively. Pd can be rapidly recruited to infection or inflammation sites, while Dd plays a role in tissue healing (56). CD209 (DC-SIGN), a C-type lectin receptor expressed on DC surfaces, is involved in bacterial recognition and antigen presentation, with higher expression in LL than other types. Dermal DCs also show similar expression patterns in TT patients. Additionally, dermal DCs are linked to matrix metalloproteinase (MMP-12) as part of a tissue remodelling network contributing to granuloma formation among TT patients (57–59). In addition, DCs have been reported to have the potential to induce anti-microbial immunity as a vaccine candidate against experimental leishmaniasis. In this study, mice inoculated with soluble leishmaniasis antigen-peptidoglycan-DC showed an increase in the expression of proinflammatory cytokines such as IL-12 and IL-17 and an attenuated effect of Leishmania protozoa-induced expression of IL-10, which suggests that the immune response is more proinflammatory (60). However, the exploration of DC application against M. leprae has not been investigated. Therefore, future studies could explore a combination of approaches, such as utilizing DCs as vaccine candidates or employing antimicrobial and anti-inflammatory strategies.

#### 3.2.2 Keratinocytes

The interaction between keratinocytes and DCs is facilitated by the intercellular adhesion molecule-1 (ICAM-1). ICAM-1 appears more abundant in TT patients than in LL. Lyrio et al. demonstrated that M. leprae interacts with keratinocytes through binding to receptors on the basal layer of the epidermis (LN-5) and the surface of keratinocytes, enhancing the expression of Cathelicidin and TNF-α (61). This provides new insights for leprosy treatment. Notably, keratinocytes were vital in two different leprosy reactions (RR/ENL), and thalidomide treatment could down-regulated the expression of ICAM-1 and HLA-DR antigens in keratinocytes. However, the increased of keratinocyte amount does not control bacterial load. The reason may be that granulocyte-macrophage colony-stimulating factor (GM-CSF) to induce an increase in keratinocytes in the skin of leprosy patients did not alter the total number of bacteria (62). Interestingly, keratinocytes may involved in the NO pathway for bacterial killing. High expression of iNOS has been observed in keratinocytes within LL granulomas compared to normal skin tissue. Furthermore, keratinocytes may upregulate HBD2 and HBD3 under leprosy stimulation, contributing to antibacterial activities (63). Wound-resident mesenchymal stromal cells can be directly reprogrammed into keratin-forming cells, offering a promising approach for treating skin damage in leprosy (64). However, rigorous studies and clinical trials are necessary to ensure safety and efficacy. Additionally, the versatility of keratinocytes, which can be reprogrammed into various cell types, highlights their potential in regenerative medicine. For instance, hair follicle stem cells (HFSCs) are keratin-forming cells in a relatively undifferentiated state. Studies have shown that transplantation of HFSCs between severed tibial nerve fragments promotes the recovery of walking ability and axonal growth in mice by forming chevron cells (65). Further exploration and analysis are crucial for realizing the clinical benefits of keratinocyte-based therapies.

#### 3.2.3 Macrophage

The cells were crucial in mediating the interactions between the host and M. leprae, with M1 and M2 types being the predominant subtypes (66). Stimulation of endothelial cells by IFN-γ or certain drugs can trigger the differentiation of monocytes into M1 macrophages through the Jagged-1 (JAG1)-dependent mechanism. In turn, it promotes the expression of TNF and IL-1, IL-6 and IL-12, leading to enhanced antimicrobial capabilities, pro-inflammatory responses, and antigen presentation (67). M2 differentiates from unstimulated endothelial cells activated by IL-4/IL-13 and exerts anti-inflammatory, fibrotic, and tissue repair effects (68–70). In TT patients, granuloma formation is dominated by M1, whereas in LL leprosy patients, M2 is predominant. Sousa et al. found that monocytes differentiate to function as M4 under the regulation of CXCL4, and the number of M4 macrophages is significantly higher in LL than in TT. In contrast, the special macrophage may be less effective in controlling M.leprae replication. In addition, IL-6, TNF-α, MRP8, MMP7 and CD68 are elevated due to M4 in TT type patients (71). Despite macrophages were known to be the primary cells involved in the host immune response to infection, the behaviour of the new subtype and its potential impact on the development of an *in situ* immune response remains unknown (72).

There are currently several bactericidal pathways in which macrophages are involved (73). To begin with, autophagy and phagocytosis are critical mechanisms for killing bacteria (74). IFN-γ induces the cytokine levels are higher in TT patients compared to LL (75). In human monocytes, the phagocytosis of M.leprae is mediated by complement receptors CR3 (76). Interaction of PGL-I with CR3 promotes bacterial invasion of macrophages (67). Second, the vitamin D-dependent bactericidal pathway also involves macrophage participation. Studies have shown that decreased gene expression of the Vitamin D Receptor gene (VDR) and Cathelicidin Antimicrobial Peptide (CAMP) gene impacts serum levels of vitamin D, cytokines, and the antimicrobial peptide Cathelicidin, thereby affecting immune pathways (77). It was reported that VDR and CAMP gene expression maintains the inflammatory response against M. leprae infection in MB and PB, even six months after MDT. Consequently, it is advisable to initiate vitamin D supplementation for leprosy patients as a transcription factor to enhance the expression of the VDR and CAMP genes (78). The study showed that in LL patients, Has-mir-21 is a mammalian micro-RNA encoded by the MIR21 gene that downregulates Toll-like receptor 2/1 heterodimer (TLR2/1) and increases IL-10 to inhibit the expression of vitamin D-dependent antimicrobial peptide (Cathelicidin) (79). At last, the iNOS-induced NO bactericidal path has gained significant attention (80). De Sousa demonstrates a positive correlation between iNOS level and cell factors like CD68, IL-22, and STAT3. RR patients show higher iNOS expression levels than patients without reaction (81). Zebrafish experiments also showed that PGL-1 induces iNOS expression, which may disrupt mitochondria, leading to demyelination, which kill leprosy bacilli and causes nerve damage (82). In addition, the mRNA and protein encoded by the S100A12 gene are induced by TLR2/1 and IFN-γ, which could directly kills bacteria. Bacteria will improve bacterial survival by up-regulating CD163 and TfR1 expression, increasing iron storage and down-regulating transporter proteins (83).

An essential concern during M. leprae infection is lipid homeostasis. There is evidence that M. leprae can induce the formation of lipid droplets (LDs) in infected macrophages, and cholesterol (Cho) is one of the host lipid molecules that accumulate in macrophages (84). Therefore, inhibition of LD formation in infected macrophages reduces bacterial survival. It was shown that M. leprae inhibits lipid degradation by suppressing hormone-sensitive lipase (HSL) expression, thereby contributing to lipid accumulation in infected macrophages (85).Clofazimine exerts its bactericidal activity on the basis of a reduction in LDL in macrophages (86). Notably, disrupting Cho metabolism by inhibiting Cho synthesis or depleting exogenous Cho with statins significantly reduced intracellular bacterial survival (87). These findings emphasise the importance of metabolic interactions between the host and the bacteria and provide a basis for identifying new pharmacological targets. These targets could control Mycobacterium infections by controlling vital metabolic pathways influencing bacterial survival.

#### 3.2.4 Schwann cells

SCs were affected by infected macrophages, which secrete TNF and its ligands or cause damage to SCs through pro-inflammatory cytokines. M.leprae binds to the alpha-2 chain of laminin-2 (LN-2) in the G domain of SCs’ basal layer through the alpha-dystroglycan (DG) protein, a process mediated by surface molecules on the cell membrane (88). The interaction activates the phosphoinositide 3-kinase (PI3K) signalling pathway, leading to the internalization of bacteria into SCs (89). Research indicates that internalization is primarily due to PGL-1 activating the Stimulator Interferon Genes (STING) cytosolic sensing pathway, recruiting infected bacteria-carrying CCR2+ monocytes to the infection site. That is why SCs exhibit a foamy phenotype after infection (82). Thus, disrupting the PGL pathway may become an essential strategy for leprosy prevention. Upon invasion of the host, M.leprae accumulates in neuroepidermal blood vessels and lymphatics and reaches neuroendothelial cells via the vascular route to infect Schwann cells (SCs). While the host expresses pro-inflammatory cytokines to destroy pathogens, T cells kill SCs *in vivo* and cause a demyelination response and inflammation (90). Studies have shown that SCs can be reprogrammed to become progenitor/stem cell-like cells (pSLC) and will promote infection into other tissues (e.g., muscle), which may also be the cause of deformities in patients (91). Inside SCs, bacteria promote their proliferation by upregulating glucose uptake and lipid synthesis while downregulating oxidative stress, apoptosis, and autophagy, similar to macrophages. Infected cells induce abnormal metabolism in SCs, leading to the accumulation of LDs. LDs are formed by the interference of CD206 and peroxisome proliferator-activated receptor-gamma (PPAR-γ) activated by peroxisome proliferator-activated receptor (PPAR) agonists, which also induces demyelination and neuroinflammatory processes (92). In addition, the presence of M. leprae in SCs seems to enhance the oxidative phases of cellular reducing power sources, including malic enzyme and the pentose phosphate pathway, through an increase in glucose uptake. Concurrently, it disrupts host metabolic processes by suppressing mitochondrial activity and significantly diminishing lactate production in infected cells (93). This finding provides a new perspective for future host-targeted therapeutic strategies against leprosy.

#### 3.2.5 Neutrophils

The role of neutrophils in leprosy has been overshadowed by numerous studies that predominantly focus on macrophages/Schwann cells in response to M. leprae. The cells could be considered the biomarker for ENL and often exhibit intense cell infiltration. It has been reported that ENL lesions exhibit a notable neutrophilic infiltrate predominantly situated within the deep layers of the dermis and subcutaneous tissue, superimposed on a background of chronic MB type. Therefore, neutrophils can be considered to some extent as markers of ENL (94, 95). However, little is known about the direct role of neutrophils in leprosy/ENL. The cells are crucial in host defence against bacteria and promote inflammation, but they are not automatically activated in TT/BL/ENL patients (96). Compared to BL/LL patients, neutrophil apoptosis is higher during the ENL (49).CD64 is expressed in patients’ neutrophil cell surface, while not in healthy individuals or non-patients. Schmitz et al. found that the severity of ENL is closely correlated with CD64 expression (97). After thalidomide treatment, CD64 expression is downregulated in neutrophils and ENL. Studies suggest that neutrophil extracellular traps (NETs) are a major source of endogenous DNA, consisting of DNA scaffolds decorated with various proteins. Excessive NETs can trigger the activation and amplification of immune-inflammatory pathways, which may be the primary pathogenic mechanism of ENL (98). In conclusion, in-depth research is needed before neutrophils can be used as a valid prognostic marker or target for ENL. In addition, we can focus on designing novel therapeutic agents, especially targeting neutrophil migration and mobilisation, to achieve precise modulation of the inflammatory process while avoiding triggering full-blown immunosuppression. This line of research is expected to provide new strategies to improve patient symptoms and optimise treatment outcomes.

### 3.3 The role of adaptive immune cells in the pathogenesis of leprosy

#### 3.3.1 Helper T cells

T helper cells are polarized into two main types (Th1/Th2). Activation of Th1 induces a shift of macrophages to an M1-polar state, and production of IFN-γ/IL-2/IL-15/TNF enhances macrophage activity in patients, predominantly TT patients. In contrast, when IL-4- and IL-10-producing Th2 are activated, they may inhibit the microbicidal function of macrophages, with patients predominantly of the LL type. It has been reported that these subsets are considered the contributors to delayed-type hypersensitivity (DTH) reactions (99). The majority of PBMCs after stimulation with M. leprae antigens showed a non-specific Th0 response, but there were also lepromatous form patients responses that showed a Th2 response, whereas tuberculoid form patients showed a Th1 cytokine response. In addition, Patients with LL have been reported to have a relative lack of CD4+ T cells in their lesions compared to TT, but a large number of CD8 T cells and macrophages are heavily infected with mycobacteria with a characteristic foamy appearance. Interestingly, in the studies from India and Brazil, Th1/Th2 cells coexist, suggesting potential bidirectional conversion between Th1/Th2 (100). Furthermore, the Notch1 signalling pathway has been found to promote lymphocyte proliferation, leading to Th cell activation and differentiation (101).

Apart from the classical Th1/Th2 subsets, other Th cell subgroups have been discovered. Compared to LL patients, TT patients exhibit higher levels of IL-9 secreted by Th9 cells (102). Studies have shown that IL-9 induces macrophage bactericidal activity in TT-type patients through collaborative responses with IFN-γ, IL-6, and IL-12. Conversely, in LL-type patients, IL-9 inhibits the production of IL-4, IFN-γ, and TNF-α (103). Th17 cells secrete cytokines that promote tissue inflammation, macrophage activation, neutrophil recruitment, and enhance Th1 response (104). Th17 cells produce signature cytokine IL-17 and transcription factors RORC and STAT3, but only tuberculoid patients have shown phosphorylated STAT3 (104). STAT3 was essential in Th cell differentiation and induction of immune memory. Th22 cells are also involved in leprosy pathogenesis, mainly secreting the fibroblast growth factor (FGF) family cytokine and cytokines such as IL-22/26. It has been reported that lepromatous form patients exhibit increased expression of FGF b, IL-13, and IL-22, while tuberculoid form patients show a more pronounced increase in TNF-α (105). Th22 has chemokine receptors such as CCR4, CCR6 and CCR10 that undergo differentiation in the presence of TNF-αand IL-6. In the lepromatous form of the disease, the Th22 response is crucial *in situ*, as FGF b can modulate various cellular functions affecting wound migration, healing, cell division, and angiogenesis process Etc. (101). In addition, IL-22 induces the production of STAT3 and iNOS to kill bacilli directly, but this is detrimental to the development of a macrophage response.

#### 3.3.2 Regulatory T cell

The Tregs are essential for maintaining host immune tolerance, limiting autoimmunity, and preventing inflammatory diseases. It is reported that Tregs can be categorized into two main subgroups: natural (CD4+ CD25+) and inducible Treg cells (106). It has been found that Treg can achieve its inhibitory effects through cytokines, with cytokines such as IL-10, IL-35, and TGF-β being of interest. Studies have shown a significant increase in the frequency of CD4+ CD25+ Tregs producing IL-35 in leprosy patients (107). The infiltration of specific cell subsets in the pathological sites is a crucial determinant of local immune response and influences the clinical presentation of the disease. Specific subgroups, including FoxP3+ Tregs, can modulate the immune response at leprosy pathological sites. Kumar et al. found that high levels of TGF-β induce the expression of FoxP3, thereby achieving immune regulation in peripheral blood (106). Analysis of IL-10-producing FoxP3+ Tregs in different polar forms and healthy controls revealed that the number of Tregs in patients was twice that of healthy contacts, and leprosy LL/BL patients also exhibited significantly higher Treg numbers. However, TGF-β in FoxP3+ Tregs might downregulate T cell responses, leading to antigen-specific hypersensitivity associated with LL. Studies have suggested that the quantity of Tregs is higher in RR patients’ PBMCs and skin lesions compared to non-reactional patients (108, 109). In LL patients, Th2/Treg polarisation appears vital for disease progression, whereas Th1/Th17 cellular immunity is essential for TT patients. Recent research suggests that levels of Tregs contribute to Th17 immune unresponsiveness in lepromatous patients. In this context, the ideal treatment for patients with LL appears to require modulation of T lymphocyte subsets to expand Th17 lymphocytes and control Treg cells, favouring the cellular immune response. Therefore, shifting the cellular immune response to Th1/Th17 may lead to better outcomes in leprosy treatment (110).

#### 3.3.3 NKT/B/γδ T cells

**Figure 2:**
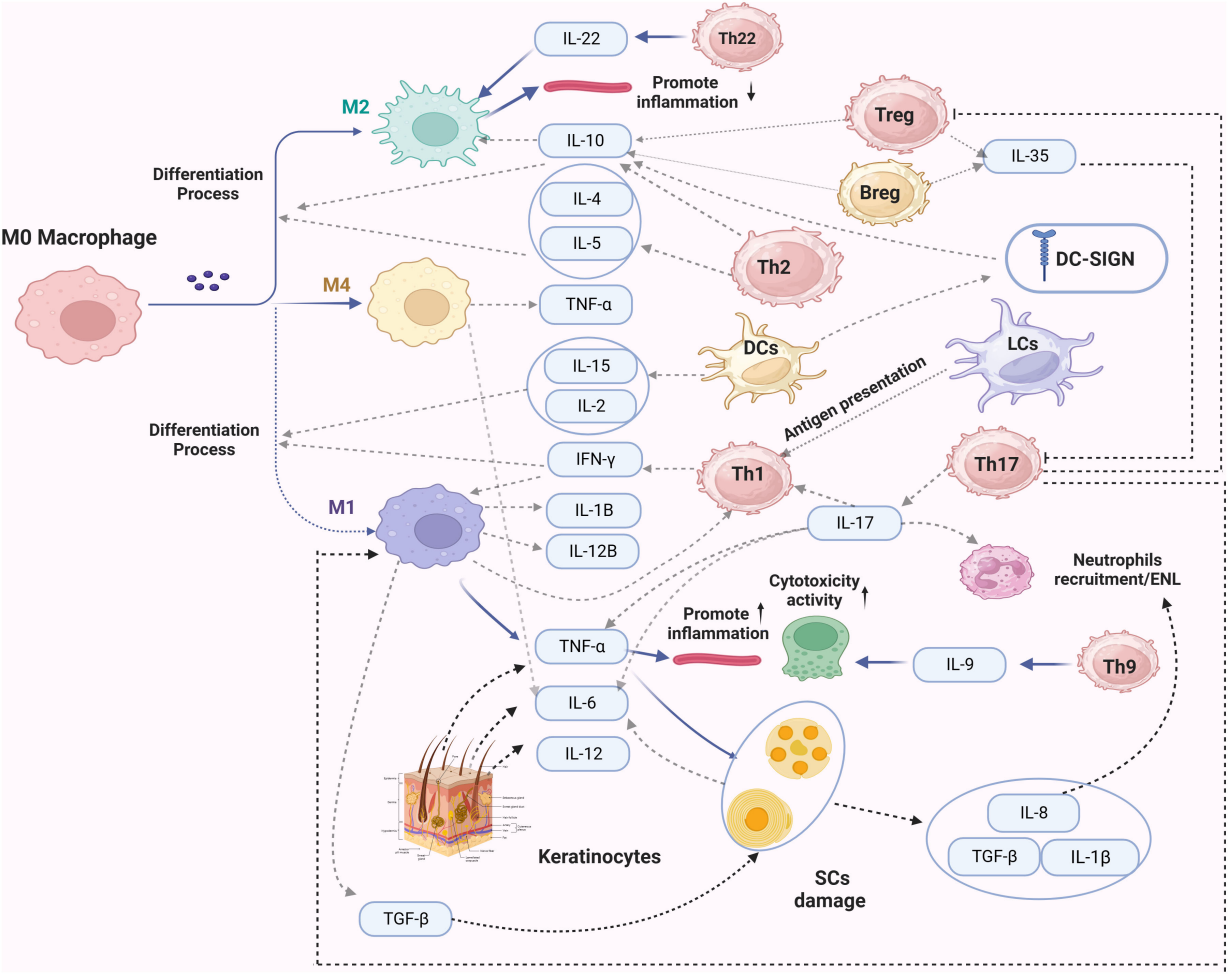
The secretion of cytokines during the immune response of leprosy infection. In this figure, dashed lines represent the primary secreted cytokines of the immune cells. Solid lines with a arrow indicate the impact of these factors on specific cells, while a solid line ending with a vertical line denotes an inhibitory effect.

There is limited research on these cells in leprosy. Evidence suggests that B-cell-mediated humoral immunity plays a weaker role in the pathogenesis, as leprosy bacilli have been detected in LL patients. In patients with a high bacterial load, there have been significant changes in the proportion of B-cell subsets (109). Studies have shown that the number of B cells is higher in ENL patients compared to non-reactional leprosy patients. Additionally, IL-10 and IL-35 expression levels are higher in Bregs, and the production of IL-10 in B-cell subsets is also higher in PBMCs (111). γδ T cells, a subset of lymphocytes, constitute a small proportion of all types of T cells. The number of γδ T cells significantly increases in leprosy granulomas, and they produce significant amounts of IL-17 and IFN-γ, which are crucial for leprosy reactions (112). Reports indicate that γδ T cells in RR/ENL patients are higher than in general patients, and they have a particular inhibitory effect on immune responses (113). NKT cells are a unique subset of mature T cells known for their rapid production of immunoregulatory cytokines upon activation. CD1d is an antigen-presenting molecule of the CD1 family. Upon recognition of antigens presented by CD1d, NKT cells quickly produce Th1/Th2 cytokines to facilitate bactericidal functions. CD1b limits the recognition of lipoarabinomannan (LAM), a purified antigen of M.leprae, thereby blocking T cell secretion of IFN-γ (69). TT patients exhibit more robust expression of the CD1 family molecules compared to LL patients. Research suggests that cytokines from NKT cells control the response of effector T cells upon LAM activation, thereby influencing overall T cell responses and clinical manifestations (114). In addition, Cytokine immunomodulation of immunological processes by Mycobacterium leprae is shown in **Figure 2**.

## 4 Leprosy is under precise regulation by genetic factors

### 4.1 Discovery of genetic predisposition genes

Evidence of the genetic susceptibility to leprosy first emerged from descriptive studies, complex segregation analysis (CSA), and twin studies related to heritability. Descriptive studies revealed familial solid clustering of leprosy cases, while CSA identified significant genetic effects controlling susceptibility and the mode of inheritance in different genetic backgrounds. Twin studies in leprosy showed higher concordance inozygotic twins than dizygotic (6). Subsequently, family-based linkage studies and association analyses were applied in leprosy research. Genome-wide linkage studies identified certain chromosomal regions (e.g., 10p13, 6q25-27) as candidate locations harbouring leprosy susceptibility genes (115). In contrast, association analysis primarily evaluated whether polymorphisms in candidate genes were associated with leprosy. In 2009, China first integrated genome-wide association studies (GWAS) with leprosy research, identifying numerous leprosy susceptibility loci (116). However, SNP-level causal associations may arise from linkage disequilibrium (LD) with adjacent alleles in the studied candidate genes. Therefore, independent replication and validation in different populations are important. Additionally, candidate gene approaches can be linked with functional studies to determine whether a gene is involved in the biological mechanisms underlying disease onset. In summary, applying these molecular strategies has identified many genes associated with leprosy, as shown in **Table 1**.

**Table 1:** The susceptibility genes of leprosy that have been replicated or functionally validated.

Chromosome position	Gene	Name	Population
1q31.3	CFH	Complement factor H	Chinese^3^ (117)
1p22.3	BCL10	B cell lymphoma 10	Chinese^3^ (118)/Brazilian^3^ (119)
1p31.3	IL23R	Interleukin 23 receptor	Chinese^1,2^ (120, 121)/Brazilian^3^ (122)/Indian^3^ (123)
1q21.3	FLG	Filaggrin	Chinese^2^ (120)
1q32.1	IL-10	Interleukin-10	Brazilian^3^ (124, 125)/Indian (126)^3^
2q12.1	IL18RAP/IL18R1	Interleukin 18 receptor accessory protein/interleukin 18 receptor 1	Chinese^3^ (127)/Vietnam (119)
3p21.31	NCKIPSD	NCK interacting protein with SH3 domain	Chinese^2^ (120)
4p14	TLR1	Toll-like receptor 1	India^1^ (128)/Brazilian^3^ (129–131)/Colombian^3^ (132)
4q31.3	TLR2	Toll-like receptor 2	Ethiopian^3^ (133)/Brazilian^3^ (85, 130)/Colombian^3^ (132)
5p14.3	CDH18	Cadherin-18	Chinese^3^ (134)
5q33.3	IL-12B	Interleukin 12B	Chinese^1^ (7)/Vietnam^1^ (135)/Indian^3^ (123, 136)
6p21.32	HLA-DR-DQ	MHC, class-II. HLA	Chinese^1^ (116)/Inida^4^ (128)/Vietnam^3^ (137)/Malawi, Mali^1^ (138)
6p21.33	HLA-C	Human Leukocyte Antigen-C	Brazilian^3^ (139)/Inida^4^ (140, 141)/Vietnam^4^ (141)
6p22.1	HLA-A	Major histocompatibility complex, class I, A	India^3^ (140)
6p21.32	HLA-DRB1	Major histocompatibility complex, class II, DR beta 1	India^3^ (128)/Chinese^1^ (142)/Brazilian, Vietnamese^3^ (143)/Argentina^3^ (144)
6p21.33	LTA	Lymphotoxin alpha	Vietnam,Brazilian,Indian^5^ (145)
6p21.33	TNFA	Tumor Necrosis Factor Alpha	Brazilian^3^ (146)
6q24.3	RAB32	RAB32, member RAS oncogene family	Chinese^1^ (120, 121)
8q21.3	RIPK2	Receptor-interacting serine/threonine kinase 2	Chinese^1^ (116)/Vietnam^3^ (137)
9q32-q33.1	TNFSF15/TNFSF8	Tumor necrosis factor (Ligand) Superfamily, Member 15, 8	Chinese^1^ (116)/Brazilian,Vietnamese^3^ (147)
9q34.3	CARD9	Caspase recruitment domain family member 9	Chinese^2^ (120)
9q34.3	FCN2	Ficolin-2	Chinese^3^ (148)/Brazilian^3^ (149, 150)
6q26	PARK2	Parkin RBR E3 Ubiquitin Protein Ligase	Vietnam,Brazilian^5^ (151)/Indian^3^ (152)
10p12.33	MRC1	Mannose receptor C-type 1	Chinese^3^ (153)/Brazilian,Vietnam^3^ (154)
10q21.1	MBL2	Manner-Binding Lectin, MBL 2	Brizilian^3^ (155, 156)/Chinese^3^ (153)/Colombia^3^ (157)
10q22.1	SLC29A3	Solute carrier family 29 member 3	Chinese^2^ (120)/Malawi, Mali^1^ (138)
12q12	LRRK2	Leucine rich repeat kinase 2/Dardarin	Chinese^1^ (116)/Inida^3^ (158)/Vietnam^3^ (159)
12q13.11	VDR	Vitamin D Receptor	Brizilian (78, 160)/India^3^ (161)
12q15	IFNG	Interferon-gamma	Chinese^1^ (153)/Brazil^3^ (162)
13q14.11	LACC1—CCDC122	Laccase domain containing—coiled-coil domain containing 122	Chinese^1^ (116, 163)/Brazilian, Vietnam^3^ (164)/ Malawi, Mali (138)
14q23.2	HIF1A	Hypoxia-Inducible Factor 1 Alpha	Chinese^2^ (134, 165)
16p12.1-p11.2	IL-27	Interleukin 27	Chinese^1^ (120)
16q12.1	NOD2	Nucleotide-binding oligomerization domain containing 2	Chinese^1^ (116)/Brazilian^3^ (166)/Vietnam^3^ (137)
19p13.2	TYK2	Tyrosine kinase 2	Chinese^2^ (120)
19q13.4	KIR(2DS1-3)	Killer immunoglobulin-like receptor	Brazil^3^ (167, 168)

The way in which susceptibility genes were first discovered: 1: GWAS, Genome-wide association study; 2: GWAS (protein-coding variants); 3. Candidate gene analysis; 4. Association scan; 5. Genome-wide linkage analysis.

### 4.2 Functionality study of susceptibility genes in leprosy immune response

#### 4.2.1 Protective defense

Loss-of-function (LoF) mutations in the FLG gene can lead to filaggrin deficiency, severely impairing the skin’s protective function. Liu Hong et al., through a whole-genome analysis of protein-coding variations, discovered an association between the functional loss mutation rs146466242 in FLG and leprosy susceptibility in Chinese (120). The study also found that individuals with post-traumatic injuries, such as tattoos or injuries caused by glass bangles, are more susceptible to leprosy. Another study identified an association between the SNP site Q1790X in FLG and disease susceptibility (169). Filaggrin deficiency can cause immune dysregulation, such as reduced IFN-γ expression in keratinocytes and increased CD11C expression in LCs. However, these mechanisms still require animal models or human skin tissue validation.

#### 4.2.2 Bacteria identification

Innate immunity is activated by recognizing pathogen-associated molecular patterns (PAMPs) and their corresponding receptors. At this stage, genetic variations can either enhance or hinder the recognition of mycobacteria, leading to varying levels of natural resistance in the host. Studies have shown genetic associations between several SNPs in TLR genes and leprosy susceptibility. For instance, the rs5433095 (N248S) variant in the TLR1 gene has been found to inhibit TLR1 signalling, contributing to disease protection, while TLR2 mutations are mainly observed in TT-type patients. However, the evidence regarding the association of SNP polymorphisms in TLR4 with susceptibility requires further investigation (170). NOD2 is also known for leprosy susceptibility among the Chinese population. The gene recognizes leprosy cell wall dipeptide (MDP), leading to the upregulation of IL-32 expression and facilitating the differentiation of monocytes into CD1b+ DCs (171). This particular pattern is more prevalent in TT patients compared to LL patients (166). Additionally, the gene MRC1 is involved in recognizing mannose residues on leprosy, and its susceptibility-related SNP rs1926736 has been validated in studies conducted in China, India, Vietnam, and Brazil (153, 154, 172). Nonetheless, further research is necessary to elucidate its functional implications.

#### 4.2.3 Antigen processing and presentation

Human Leukocyte Antigen (HLA) plays a pivotal role in the adaptive immune response and is essential for antigen presentation to T cells. Enhancing disease diagnosis and treatment requires pinpointing the most clinically significant HLA variants. However, genetic diversity within and between populations poses a challenge for HLA genomics to become a standard component of health care. Improving the diagnosis of human diseases and treatment options requires the identification of the HLA variants that are most clinically relevant. However, genetic diversity within and between populations poses a challenge to the realization of this idea. The class I and II genes have already been demonstrated as the leprosy susceptibility genes. It was found that the class I gene HLA-A28 was notably linked to a heightened risk of leprosy among Mexican Mestizos. Carriers of this SNP demonstrated 2.12 and 2.74 times greater likelihood of developing leprosy and lepromatous subtypes (173). HLA-C12, HLA-B15, HLA-C05 was associated with Brazilian patients (139, 174). HLA-A, HLA-C and HLA-Cw may associated with the Indian population (140). It has been reported that leprosy-associated class II HLA genes include HLA-DRB1 in Vietnamese (143, 175), HLA-DQA1/HLA-DPB1 in Brazilian, HLA-DQB1 in Mestizo (173), HLA-DRB1 in Argentinean (144), HLA-DRB1/HLA-DR-DQ in Chinese (116, 142, 176), and HLA-DQA1/HLA-DRB1 in Indian (128). These susceptibility genes of HLA are also associated with leprosy complications. Notably, screening of HLA-B13:01-positive patients has shown that the discontinuation of dapsone intake can significantly eliminate dapsone hypersensitivity syndrome (DHS), and this result has also been validated in Thai patients (175). The interaction between HLA-B13:01 and T cell activation stands out as a crucial element in the initiation of DHS. The identification of individuals at risk for DHS becomes feasible through testing for the presence of HLA-B13:01 and TCR clones. In addition, the methods allow for a more precise and effective screening process, enabling timely interventions and personalized medical strategies for those susceptible to DHS (119). Moreover, five amino acid variants in HLA-DRB1 are also closely related to the DHS in Chinese patients. Recent research indicates that the four amino acid polymorphisms in HLA-DRB1, HLA-B, and HLA-A were recently identified as key factors in the association of leprosy with HLA in Vietnamese cases. This discovery paves the way for focused protein-HLA peptide binding studies and reduces the interference of linkage disequilibrium (LD) with the associations (175). A meta-analysis also showed that variants in HLA-DRB1, DQA1, and HLA-C were associated with the Chinese population (176). In addition, HLA-G, MICA, or TAP1 have also been suggested to be possibly associated with antigen presentation, but their roles need to be further investigated (177–179).

#### 4.2.4 Autophagy and phagocytosis

Several susceptibility genes related to autophagy and phagocytosis have been identified, including RAB32, LRRK2, and PARK2. In addition to their association with leprosy, RAB32 susceptibility has also been linked to PD and CD. RAB32 regulates autophagy, phagocytosis, and mitochondrial functions in PD, suggesting a similar role in leprosy. LRRK2 was first discovered in a GWAS of the Chinese population and subsequently replicated in Indian, Brazilian, and Vietnamese populations (158, 180). Cellular experiments confirmed the contribution of NOD2 in the immune response in leprosy. Mutations in LRRK2 and NOD2 genes result in abnormal interactions affecting host antimicrobial responses, inhibiting NOD2 signal transduction and causing ROS accumulation. Interestingly, PD patients with overexpressed constitutively active Rab32 may exhibit decreased mitochondrial LRRK2 content. The SNP in LRRK2 is also closely related to RR. It has been hypothesised that the R1628P-LRRK2 kinase mutation may prevent RR by reducing apoptosis and releasing anti-inflammatory mediators, thereby reducing apoptotic debris production (181, 182). Therefore, drug development against LRRK2 as a therapeutic target is potentially possible. The association of the PARK2 gene with leprosy susceptibility was discovered through positional cloning and is linked to polymorphisms in the upstream regulatory region of PACRG (180). Loss-of-function mutations in PARK2 impair the interaction between the protein and E2 ligase and its protein substrates. However, this association has yet to be found in the Chinese population. Furthermore, the research group has identified SLC29A3 as a susceptibility gene for leprosy, which encodes equilibrative nucleoside transporter 3 (ENT3). Studies have shown that the lack of ENT3 leads to enlargement and disruption of lysosomal compartments, accumulating residual mitochondria, increased intracellular ROS, and DNA damage in T cells (138). The IRGM gene, which regulates autophagy, has also been confirmed to be associated with leprosy susceptibility; it was demonstrated that only rs13361189 TC and CC genotypes are significantly associated with leprosy (75).

#### 4.2.5 Cytotoxic genes

Several susceptibility genes related to microbial killing have been identified. As previously mentioned, the VDR gene mediates the transcription of various antimicrobial peptides to achieve bactericidal effects. Functional SNPs in the gene can influence the balance of the vitamin D pathway, including FokI (rs2228570), TaqI (rs731236), ApaI (rs7975232), and Bsm I (rs1544410) (183). The amount of NO synthesized by macrophages depends on L-arginine availability, which is closely related to enhanced mRNA expression of SLC7A2, the gene encoding the transport activation of L-arginine. Recent research suggests that the OPA1 gene may also be involved in the host’s mitochondrial antibacterial mechanisms. Two SNPs (rs9838374 and rs9838374) of the gene have been linked to leprosy susceptibility in China (116). The polymorphism of LACC1 (C13orf31) influences the production of mitochondria and NADPH oxidase-dependent ROS in macrophages, affecting bactericidal activity and inflammasome activation (116). Additionally, genes encoding bactericidal proteins, such as cathepsin B (CTSB), beta-defensin 1 (DEFB1), and interferon-gamma (IFNG), have also been confirmed. Furthermore, The activation of KIR (KIR2DS1, 2DS2, and 3DS1) and their associations with HLA ligands are related to leprosy in the Brazilian population (184). In summary, further research is still required on these genes.

#### 4.2.6 Functional genes in the complement system

The complement system plays a crucial role in the host’s innate immune processes, as it activates and regulates protein hydrolysis cascades and pro-inflammatory responses. Several genes involved in the complement system have been confirmed to be associated with leprosy susceptibility. These genes include Mannose-Binding Lectin (MBL), Ficolins (FCN), Complement Receptor 1 (CR1/CD35), and Complement Factor H (CFH) (149). MBL2 binds with bacteria, enhancing the phagocytosis of M.leprae by macrophages *in vitro*. Research has shown that polymorphisms in the exons and promoter region of the MBL2 gene confer susceptibility to leprosy in Brazil (149, 185). Another GWAS study has confirmed the association of complement genes FCN2, MBL2, and CFH with leprosy susceptibility (148). FCN2 is a soluble pattern recognition molecule that can bind to different pathogen-associated PAMPs, triggering phagocytosis and activating the complement pathway through the MBL pathway (186). Haplotypes and genotypes with MBL deficiencies can prevent the progression of leprosy to lepromatous leprosy (187). Based on this, relevant therapeutic and prevention strategies may include enhancing the activity of FCN2 to improve the recognition and clearance of leprosy pathogens. Additionally, interventions addressing MBL deficiencies could be explored to prevent leprosy from advancing to a more severe form. Moreover, Complement Receptor 1 (CR1) can mediate the entry of M. leprae into phagocytic cells, and its association with leprosy susceptibility has been confirmed in populations from Malawi and Brazil. However, unfortunately, this association has not been found in the Chinese population.

### 4.3 Genes associated with cytokines

Cytokines have been the focus of genetic and immunological research. The association of IL-10 (rs1800871) with leprosy has been firmly established. Studies in Brazil have revealed significant associations between leprosy susceptibility and the SNPs rs1800872/rs1800896 (188). The widely studied TNF variant rs1800629 has been validated in different populations. Interestingly, a meta-analysis showed an association between rs1800629 and leprosy in Latin American populations but not in Asian populations. The understanding of Lymphotoxin-alpha (LTA-α) remains limited. Initial studies using linkage disequilibrium mapping identified a significant association between the LTA+80 A variant polymorphism and leprosy (145). A systematic scan of the BAT1-LTA-TNF-BTNL2 region also found a connection between LTA/TNF and leprosy, with results verified in populations from India, Vietnam, and Brazil (189). These two cytokines and IFNG are associated with leprosy granuloma formation. IFNG, responsible for secreting interferon-gamma (IFN-γ), activates innate immune cells. The rs2430561 was linked to elevated serum IFN-γ levels in healthy individuals and TT leprosy patients. Nevertheless, gene expression profiling of leprosy skin lesions has revealed that IL-27 inhibits IFN-γ activity, consequently dampening the antimicrobial response of the host (190). Therefore, blocking IL-27 could inhibit the immunosuppressive pathway induced by IFN-β while preserving its immunostimulatory function. Notably, focusing on IL-27 as a therapeutic target could be utilized as a complementary approach alongside conventional antibiotic therapy.

### 4.4 Genes associated with leprosy reactions

Susceptibility genes can control the clinical manifestations of patients and are strongly associated with the development of leprosy reactions. One study examined the correlation between the TNFSF15/TNFSF8 locus and RR in ENL and found that only TNFSF8 was genetically correlated with RR. Among them, TNFSF15 mediates the transition from Th1 to Th2 phenotype. In the Vietnamese population, the TNFSF15/TNFSF8 variants rs6478108 and rs7863183 were associated with RR, which was not observed in Brazilian patients. After excluding age, the study found that rs3181348 was also a risk factor for RR (191). Thus, TNFSF8 may mediate excessive inflammation, and transcript levels are age-related. rs6807915 near SYN2 was identified as a susceptibility locus for leprosy by Liu et al. (192). It was hypothesized that the autophosphorylated proteins encoded by SYN2 might be a risk factor for nerve damage. In this study, BBS9, MED20, and CTSB were also identified as leprosy susceptibility genes. However, their role in leprosy remains unknown. In addition, several genes associated with susceptibility to leprosy reactions have been identified. For example, HLA genes (e.g., HLA-B15) are associated with RR, and aldo-keto reductase family 1 member B10 (AKR1B10) is also expressed in patients with ENL and may be a potential marker or target of the leprosy response. Recent studies suggest that the drugs that inhibit AKR1B10 may effectively prevent the onset of ENL and reduce the intensity and frequency. This suggests that the development of anti-AKR1B10 drugs could help mitigate the side effects of traditional drugs such as thalidomide (Glaucoma, diabetes, obesity, etc.) (193). Polymorphisms in IL-8 and IL-17A were associated with RR responses in a southern Brazilian population (194). In Brazil, the 274 C/T polymorphism of the NRAMP1 gene may be helpful in determining susceptibility to type II reactions in leprosy patients. At the same time, it has also been suggested that the gene may be associated with drug resistance. Furthermore, elevated levels of IL-6 during ENL have been documented (109, 195, 196). In summary, limited by the specificity of M.lepare, more studies on the genetic mechanisms of the leprosy response are needed.

It is noteworthing that many genes expressed in cells and tissues from leprosy lesions have been identified (197–199). Their role in disease susceptibility and progression remains largely unknown. Many of these genes are associated with the regulation of the immune system in leprosy. On the other hand, many of these genes are also differentially expressed in numerous other diseases, particularly autoimmune and neoplastic diseases. Some authors have warned that the progression of leprosy presents strategies similar to those observed in neoplasms. This is a practically unexplored field in leprosy and perhaps many drugs developed to combat these deregulated genes in neoplasms could also be used in leprosy. As an example, AKR1B10 is a gene overexpressed in different neoplasms, with special importance in pancreatic duct carcinoma, but also expressed in leprosy reactions.

### 4.5 Susceptibility genes associated with cellular stress and inflammation

Specific susceptibility genes also play a role in host oxidative stress, lipid metabolism, and ubiquitin-mediated processes have been observed. Studies have found that the oxidative stress genes HIF1A and SOD2 are associated with susceptibility. The protein encoded by SOD2 regulates reactive oxygen species (ROS) levels to maintain oxidative balance and alleviate damage (200). The polymorphic site rs295340 has been confirmed as a susceptibility locus in Brazilian patients. HIF1A, which encodes hypoxia-inducible factor 1-alpha (HIF1-α) protein, is essential in oxygen homeostasis in cellular environments. The variant rs142179458 of HIF1A is associated with leprosy susceptibility in the Chinese population. Recently, the mitochondrial ribosomal protein MRPS5 gene (rs200730619) has also been found to be associated with leprosy susceptibility (165). As previously mentioned, the accumulation of lipids within macrophages may favour bacterial survival. Studies have identified APOE and ALDH2 genes related to lipid formation and susceptibility genes for leprosy. The locus rs405509/rs7412 on ApoE and rs671 on ALDH2 show higher associations in the Chinese population (201).

## 5 Discussion

In recent years, conducting large-scale macroscopic studies of leprosy has become extremely difficult. Priority should be directed to genetics and immunology studies in the future. The comprehensive studies not only aid in pinpointing potential therapeutic targets but also facilitate the development of more precise strategies to enable early diagnosis and prevention.

Based on the current knowledge, the innate immune plays a more critical role in initiating neurological damage and influences the initial manifestations of leprosy. However, this hypothesis needs to be further tested, especially among HHCs. The potential of innate immune cells as therapeutic targets is widely recognised but also needs to be further explored by new technologies, such as single-cell sequencing and spatial transcriptomes. Macrophages in tissues have been extensively studied, but the phenotype, functional characteristics, and interactions with the cutaneous sensory nervous system of macrophages in the skin still need to be clarified. Notably, M. leprae primarily exploits vulnerabilities in the immune response to achieve an escape process, especially the macrophage and VDR pathways. Proteins or genes in these pathways are the most promising targets for immunoprophylaxis and drug development. Furthermore, emerging evidence suggests the role of epigenetic modifications in M. leprae-infected host cells as potential contributors to disease susceptibility. Evaluating the epigenetic landscape of SCs and macrophages in response to M. leprae allows us to establish connections between genetic variation, genomics, and the environment. For instance, alterations in DNA methylation can either bolster the host’s protective immunity to eliminate the pathogen or assist the pathogen in evading the host’s immune response and persisting within the host (202). Regarding adaptive immunity, Treg, NKT, and Th17 cells will be vital in achieving disease control. Other adaptive immune cell subsets, such as B, Th9, and γδ T cells, have also been identified in patients, but the exact role has not been determined to date. Further investigations could therefore be centred around the areas mentioned above that have not yet been fully explored. In summary, DCs, Keratinocytes, Macrophages, and SCs can be parasitized by M leprae and have their functions modified by the M. leprae. In BL and LL patients, parasitism of these cells is common. Parasitism by M. leprae in lymphocytes and neutrophils has not been observed. Furthermore, adipocytes, fibroblasts, smooth muscle cells, and endothelium are also parasitized by M. leprae.

There is an urgent need to develop reliable and accurate diagnostic tools for all leprosy patients. More cost-effective, reliable and rapid diagnostic tests should be prioritized. However, achieving large-scale genetic testing for leprosy still needs to be improved. There are several reasons for this. Firstly, although previous GWAS studies have identified many genetic variants associated with leprosy, their impact on disease risk tends to be minor, explaining only a tiny fraction of the phenotypic variation. Moreover, there needs to be more investigation into genomic variations beyond SNP exploration in leprosy. Structural variants, such as extended deletions or duplications, could contribute to leprosy susceptibility through gene dosage effects. Future studies with large samples are crucial to improve the statistical power of variant association analyses. Secondly, there may be heterogeneity in genetic effects across racial populations. For example, genetic variants in SNPs in the PARK2 and PACRG genes were associated with patients from Vietnam and Brazil. However, these associations were not observed in Chinese and Indian patients. This may be due to differences in polymorphisms or genes contributing to disease risk in different ethnic groups, variability in epistatic interactions or permeability, and cascading imbalances in specific populations. Thirdly, population stratification between cases and controls was not considered. At the same time, extensive characterization of cases will be needed to reduce phenotypic heterogeneity. Factors such as polarity type and gender may improve the success of rare variant analyses. For instance, the higher incidence of male patients than females suggests a broader influence of behavioural and social factors. However, the studies of genetic variants’ impact on chromosomes in this context remain relatively unexplored. Lastly, there may be differences in the categorization of leprosy cases, and these factors need to be carefully considered in study design and analyses.

In summary, prevention and early diagnosis are the most effective strategies for eliminating leprosy. Investing in vaccine research (LepVax/MiP. et al.) and strengthening coordination between tuberculosis and leprosy are essential. In addition, developing a leprosy-specific vaccine that promotes a durable T-cell response is also a research goal. However, the BCG vaccine remains the sole available option. The inability to culture M. leprae *in vitro* hinders the exploration process, including studying drug resistance mechanisms. Fortunately, rifapentine based on SDR-PEP has a very high protective efficacy as a chemoprophylactic agent and should be further promoted in leprosy-endemic countries. Moreover, current diagnosis primarily relies on the recognition of signs and symptoms, and delays in diagnosis are common, increasing the risk of severe disability. Combining humoral markers for capturing MB patients and cellular markers for detecting PB patients has significantly improved detection rates. Therefore, the ongoing focus on identifying potential markers remains crucial for advancing early diagnosis efforts.

## Author contributions

XL: Conceptualization, Methodology, Writing – original draft, Writing – review & editing. YM: Conceptualization, Investigation, Writing – review & editing. GJ: Conceptualization, Project administration, Supervision, Writing – review & editing. GL: Methodology, Supervision, Visualization, Writing – review & editing. LX: Conceptualization, Writing – review & editing. YL: Funding acquisition, Writing – review & editing. PW: Conceptualization, Methodology, Project administration, Supervision, Writing – review & editing. LZ: Funding acquisition, Methodology, Resources, Supervision, Visualization, Writing – review & editing.

## References

[1] Fava VM Dallmann-Sauer M Schurr E. Genetics of leprosy: today and beyond. Hum Genet (2020) 139:835–46. doi: 10.1007/s00439-019-02087-5 31713021

[2] WHO. Global leprosy (Hansen disease) update, 2019: time to step-up prevention initiatives. Wkly Epidemiol Rec (2020) 95:417–40.

[3] Alemu BW Naafs B. Position statement: LEPROSY: Diagnosis, treatment and follow-up. J Eur Acad Dermatol (2019) 33:1205–13. doi: 10.1111/jdv.15569 30945360

[4] Gaschignard J Grant AV Thuc NV Orlova M Cobat A Huong NT. Pauci- and multibacillary leprosy: two distinct, genetically neglected diseases. PloS Negl Trop D (2016) 10:e4345. doi: 10.1371/journal.pntd.0004345 PMC4878860 27219008

[5] Wong SH Hill AV Vannberg FO. Genomewide association study of leprosy. N Engl J Med (2010) 362:1446–7, 1447-8. doi: 10.1056/NEJMc1001451 20393182

[6] Misch EA Berrington WR Vary JJ Hawn TR. Leprosy and the human genome. Microbiol Mol Biol R (2010) 74:589–620. doi: 10.1128/MMBR.00025-10 PMC3008172 21119019

[7] Schurr E Gros P. A common genetic fingerprint in leprosy and Crohn’s disease? N Engl J Med (2009) 361:2666–8. doi: 10.1056/NEJMe0910690 20018963

[8] Greggio E Civiero L Bisaglia M Bubacco L. Parkinson’s disease and immune system: is the culprit LRRKing in the periphery? J Neuroinflamm (2012) 9:94. doi: 10.1186/1742-2094-9-94 PMC3391996 22594666

[9] Kar HK Gupta R. Treatment of leprosy. Clin Dermatol (2015) 33:55–65. doi: 10.1016/j.clindermatol.2014.07.007 25432811

[10] Aubry A Sammarco RP Chauffour A Fletcher ML Cambau E Avanzi C. Drug resistance in leprosy: An update following 70years of chemotherapy. Infect Dis Now (2022) 52:243–51. doi: 10.1016/j.idnow.2022.04.001 35483633

[11] Scollard DM Adams LB Gillis TP Krahenbuhl JL Truman RW Williams DL. The continuing challenges of leprosy. Clin Microbiol Rev (2006) 19:338–81. doi: 10.1128/CMR.19.2.338-381.2006 PMC1471987 16614253

[12] Ogunsumi DO Lal V Puchner KP van Brakel W Schwienhorst-Stich EM Kasang C. Measuring endemicity and burden of leprosy across countries and regions: A systematic review and Delphi survey. PloS Negl Trop D (2021) 15:e9769. doi: 10.1371/journal.pntd.0009769 PMC8483296 34543282

[13] Schreuder PA Noto S Richardus JH. Epidemiologic trends of leprosy for the 21st century. Clin Dermatol (2016) 34:24–31. doi: 10.1016/j.clindermatol.2015.11.001 26773620

[14] Smith CS Aerts A Saunderson P Kawuma J Kita E Virmond M. Multidrug therapy for leprosy: a game changer on the path to elimination. Lancet Infect Dis (2017) 17:e293-7. doi: 10.1016/S1473-3099(17)30418-8 28693853

[15] Global leprosy (Hansen disease) update, 2022: new paradigm – control to elimination Geneva: World Health Organization (2023) p. 409–30.

[16] Cole ST Eiglmeier K Parkhill J James KD Thomson NR Wheeler PR. Massive gene decay in the leprosy bacillus. Nature (2001) 409:1007–11. doi: 10.1038/35059006 11234002

[17] Miftahussurur M Shrestha PK Subsomwong P Sharma RP Yamaoka Y. Emerging Helicobacter pylori levofloxacin resistance and novel genetic mutation in Nepal. BMC Microbiol (2016) 16:256. doi: 10.1186/s12866-016-0873-6 27809767 PMC5096319

[18] Williams DL Gillis TP. Drug-resistant leprosy: monitoring and current status. Leprosy Rev (2012) 83:269–81. doi: 10.47276/lr.83.3.269 23356028

[19] Meier A Heifets L Wallace RJ Zhang Y Brown BA Sander P. Molecular mechanisms of clarithromycin resistance in Mycobacterium avium: observation of multiple 23S rDNA mutations in a clonal population. J Infect Dis (1996) 174:354–60. doi: 10.1093/infdis/174.2.354 8699066

[20] Swain SS Paidesetty SK Dehury B Sahoo J Vedithi SC Mahapatra N. Molecular docking and simulation study for synthesis of alternative dapsone derivative as a newer antileprosy drug in multidrug therapy. J Cell Biochem (2018) 119:9838–52. doi: 10.1002/jcb.27304 30125973

[21] Khan S Punnoose K Bishara N Ali R Khan S Ahmad S. Identification of potential inhibitor molecule against MabA protein of Mycobacterium leprae by integrated in silico approach. J Biomol Struct Dyn (2023) 41(20), 11231–11246. doi: 10.1080/07391102.2022.2160818 36661253

[22] Cambau E Saunderson P Matsuoka M Cole ST Kai M Suffys P. Antimicrobial resistance in leprosy: results of the first prospective open survey conducted by a WHO surveillance network for the period 2009-15. Clin Microbiol Infect (2018) 24:1305–10. doi: 10.1016/j.cmi.2018.02.022 PMC6286419 29496597

[23] Liu D Zhang Q Sun Y Wang C Zhang Y Fu X. Drug resistance in Mycobacterium leprae from patients with leprosy in China. Clin Exp Dermatol (2015) 40:908–11. doi: 10.1111/ced.12665 25991507

[24] Riccardi N Giacomelli A Canetti D Comelli A Intini E Gaiera G. Clofazimine: an old drug for never-ending diseases. Future Microbiol (2020) 15:557–66. doi: 10.2217/fmb-2019-0231 32476494

[25] Neelan PN Noordeen SK Sivaprasad N. Chemoprophylaxis against leprosy with acedapsone. Indian J Med Res (1983) 78:307–13. 6674153

[26] Noordeen SK Neelan PN Munaf A. Chemoprophylaxis against leprosy with acedapsone. interim Rep Lepr India (1980) 52:97–103. 6991817

[27] Russell DA Worth RM Scott GC Vincin DR Jano B Fasal P. Experience with acedapsone (DADDS) in the therapeutic trial in New Guinea and the chemoprophylactic trial in Micronesia. Int J Lepr Other Mycobact Dis (1976) 44:170–6. 776853

[28] Lew J Kim YS. Chemoprophylaxis of leprosy contacts with D.D.S. Yonsei Med J (1966) 7:47–51. doi: 10.3349/ymj.1966.7.1.47 5974426

[29] Smith CM Smith WC. Chemoprophylaxis is effective in the prevention of leprosy in endemic countries: a systematic review and meta-analysis. MILEP2 Study Group. Mucosal Immunology of Leprosy. J Infect (2000) 41:137–42. doi: 10.1053/jinf.2000.0698 11023757

[30] Neelan PN Sirumban P Sivaprasad N. Limited duration acedapsone prophylaxis in leprosy. Indian J Lepr (1986) 58:251–6. 3543161

[31] Richardus JH Tiwari A Barth-Jaeggi T Arif MA Banstola NL Baskota R. Leprosy post-exposure prophylaxis with single-dose rifampicin (LPEP): an international feasibility programme. Lancet Glob Health (2021) 9:e81–90. doi: 10.1016/S2214-109X(20)30396-X 33129378

[32] Tawfik GM Biala M Yousef YM Tiwari R Dobs M Lotfy CI. Efficacy of chemoprophylaxis and immunoprophylaxis in leprosy prevention: a systematic review and network meta-analysis of randomized controlled trials. Clin Microbiol Infect (2021) 27:1754–61. doi: 10.1016/j.cmi.2021.07.032 34332107

[33] Mieras L Anthony R van Brakel W Bratschi MW van den Broek J Cambau E. Negligible risk of inducing resistance in Mycobacterium tuberculosis with single-dose rifampicin as post-exposure prophylaxis for leprosy. Infect Dis Poverty (2016) 5:46. doi: 10.1186/s40249-016-0140-y 27268059 PMC4897814

[34] Richardus RA Butlin CR Alam K Kundu K Geluk A Richardus JH. Clinical manifestations of leprosy after BCG vaccination: an observational study in Bangladesh. Vaccine (2015) 33:1562–7. doi: 10.1016/j.vaccine.2015.02.017 25701674

[35] Mowla MR Ara S Mizanur RA Tripura SP Paul S. Leprosy reactions in postelimination stage: the Bangladesh experience. J Eur Acad Dermatol (2017) 31:705–11. doi: 10.1111/jdv.14049 27859670

[36] Ter Ellen F Tielens K Fenenga C Mieras L Schoenmakers A Arif MA. Implementation approaches for leprosy prevention with single-dose rifampicin: A support tool for decision making. PloS Negl Trop D (2022) 16:e10792. doi: 10.1371/journal.pntd.0010792 PMC9612816 36251696

[37] Richardus R Alam K Kundu K Chandra RJ Zafar T Chowdhury AS. Effectiveness of single-dose rifampicin after BCG vaccination to prevent leprosy in close contacts of patients with newly diagnosed leprosy: A cluster randomized controlled trial. Int J Infect Dis (2019) 88:65–72. doi: 10.1016/j.ijid.2019.08.035 31499206

[38] Mieras LF Taal AT van Brakel WH Cambau E Saunderson PR Smith W. An enhanced regimen as post-exposure chemoprophylaxis for leprosy: PEP+. BMC Infect Dis (2018) 18:506. doi: 10.1186/s12879-018-3402-4 30290790 PMC6173927

[39] Wang L Wang H Yan L Yu M Yang J Li J. Single-dose rifapentine in household contacts of patients with leprosy. N Engl J Med (2023) 388:1843–52. doi: 10.1056/NEJMoa2205487 37195940

[40] World HO. BCG vaccine: WHO position paper, February 2018 - Recommendations. Vaccine (2018) 36:3408–10. doi: 10.1016/j.vaccine.2018.03.009 29609965

[41] Gomes RR Antunes DE Dos SD Sabino E Oliveira DB Goulart I. and leprosy household contacts: Protective effect and probability to becoming sick during follow-up. Vaccine (2019) 37:6510–7. doi: 10.1016/j.vaccine.2019.08.067 31500969

[42] Lwin K Sundaresan T Gyi MM Bechelli LM Tamondong C Garbajosa PG. BCG vaccination of children against leprosy: fourteen-year findings of the trial in Burma. B World Health Organ (1985) 63:1069–78. PMC2536472 2940028

[43] Richardus RA Alam K Pahan D Feenstra SG Geluk A Richardus JH. The combined effect of chemoprophylaxis with single dose rifampicin and immunoprophylaxis with BCG to prevent leprosy in contacts of newly diagnosed leprosy cases: a cluster randomized controlled trial (MALTALEP study). BMC Infect Dis (2013) 13:456. doi: 10.1186/1471-2334-13-456 24088534 PMC3850918

[44] Schoenmakers A Mieras L Budiawan T van Brakel WH. The state of affairs in post-exposure leprosy prevention: A descriptive meta-analysis on immuno- and chemo-prophylaxis. Res Rep Trop Med (2020) 11:97–117. doi: 10.2147/RRTM.S190300 33117053 PMC7573302

[45] Duthie MS Pena MT Ebenezer GJ Gillis TP Sharma R Cunningham K. LepVax, a defined subunit vaccine that provides effective pre-exposure and post-exposure prophylaxis of M. leprae infection. NPJ Vaccines (2018) 3:12. doi: 10.1038/s41541-018-0050-z 29619252 PMC5871809

[46] Muniyandi M Singh M Singh M Rajshekhar K Katoch K. Cost-effectiveness of incorporating Mycobacterium indicus pranii vaccine to multidrug therapy in newly diagnosed leprosy cases for better treatment outcomes & immunoprophylaxis in contacts as leprosy control measures for National Leprosy Eradication Programme in India. Indian J Med Res (2021) 154:121–31. doi: 10.4103/ijmr.IJMR_661_20 PMC8715682 34782538

[47] Kamath S Vaccaro SA Rea TH Ochoa MT. Recognizing and managing the immunologic reactions in leprosy. J Am Acad Dermatol (2014) 71:795–803. doi: 10.1016/j.jaad.2014.03.034 24767732

[48] Upputuri B Pallapati MS Tarwater P Srikantam A. Thalidomide in the treatment of erythema nodosum leprosum (ENL) in an outpatient setting: A five-year retrospective analysis from a leprosy referral centre in India. PloS Negl Trop D (2020) 14:e8678. doi: 10.1371/journal.pntd.0008678 PMC7577491 33035210

[49] Oliveira RB Moraes MO Oliveira EB Sarno EN Nery JA Sampaio EP. Neutrophils isolated from leprosy patients release TNF-alpha and exhibit accelerated apoptosis in vitro. J Leukocyte Biol (1999) 65:364–71. doi: 10.1002/jlb.65.3.364 10080541

[50] Tajalli M Wambier CG. Lucio’s phenomenon. N Engl J Med (2021) 384:1646. doi: 10.1056/NEJMicm2025081 33913641

[51] Gutierrez-Villarreal IM Ocampo-Candiani J Villarreal-Martinez A Gomez-Flores M Fernandez LT Rodriguez-Tamez G. Leprosy reactions after SARS-COV2 (COVID-19) infection. J Eur Acad Dermatol (2023) 37:e952–e954. doi: 10.1111/jdv.19103 37016975

[52] BROWNE SG DAVIS EM. Reaction in leprosy precipitated by smallpox vaccination. Leprosy Rev (1962) 33:252–4. doi: 10.5935/0305-7518.19620028 14016077

[53] Hunger RE Sieling PA Ochoa MT Sugaya M Burdick AE Rea TH. Langerhans cells utilize CD1a and langerin to efficiently present nonpeptide antigens to T cells. J Clin Invest (2004) 113:701–8. doi: 10.1172/JCI200419655 PMC351318 14991068

[54] Hirai KE Aarao TL Silva LM de Sousa JR de Souza J Dias LJ. Langerhans cells (CD1a and CD207), dermal dendrocytes (FXIIIa) and plasmacytoid dendritic cells (CD123) in skin lesions of leprosy patients. Microb Pathog (2016) 91:18–25. doi: 10.1016/j.micpath.2015.11.013 26639680

[55] de Oliveira AL Amadeu TP de Franca GA Menezes VM Da CNJ Pinheiro RO. Role of CD8(+) T cells in triggering reversal reaction in HIV/leprosy patients. Immunology (2013) 140:47–60. doi: 10.1111/imm.12108 23566249 PMC3809705

[56] Simoes QJ de Oliveira MF Ribeiro GA de Brito EB de Brito RB Pagliari C. CD1a and factor XIIIa immunohistochemistry in leprosy: a possible role of dendritic cells in the pathogenesis of Mycobacterium leprae infection. Am J Dermatopath (2009) 31:527–31. doi: 10.1097/DAD.0b013e31819f1997 19590423

[57] Inkeles MS Teles RM Pouldar D Andrade PR Madigan CA Lopez D. Cell-type deconvolution with immune pathways identifies gene networks of host defense and immunopathology in leprosy. JCI Insight (2016) 1:e88843. doi: 10.1172/jci.insight.88843 27699251 PMC5033757

[58] Krutzik SR Tan B Li H Ochoa MT Liu PT Sharfstein SE. TLR activation triggers the rapid differentiation of monocytes into macrophages and dendritic cells. Nat Med (2005) 11:653–60. doi: 10.1038/nm1246 PMC1409736 15880118

[59] Sieling PA Chatterjee D Porcelli SA Prigozy TI Mazzaccaro RJ Soriano T. CD1-restricted T cell recognition of microbial lipoglycan antigens. Science (1995) 269:227–30. doi: 10.1126/science.7542404 7542404

[60] Jawed JJ Majumder S Bandyopadhyay S Biswas S Parveen S Majumdar S. SLA-PGN-primed dendritic cell-based vaccination induces Th17-mediated protective immunity against experimental visceral leishmaniasis: a crucial role of PKCbeta. Pathog Dis (2016) 74:ftw041. doi: 10.1093/femspd/ftw041 27150838

[61] Lyrio EC Campos-Souza IC Correa LC Lechuga GC Vericimo M Castro HC. Interaction of Mycobacterium leprae with the HaCaT human keratinocyte cell line: new frontiers in the cellular immunology of leprosy. Exp Dermatol (2015) 24:536–42. doi: 10.1111/exd.12714 25828729

[62] Kaplan G Walsh G Guido LS Meyn P Burkhardt RA Abalos RM. Novel responses of human skin to intradermal recombinant granulocyte/macrophage-colony-stimulating factor: Langerhans cell recruitment, keratinocyte growth, and enhanced wound healing. J Exp Med (1992) 175:1717–28. doi: 10.1084/jem.175.6.1717 PMC2119267 1588289

[63] Cogen AL Walker SL Roberts CH Hagge DA Neupane KD Khadge S. Human beta-defensin 3 is up-regulated in cutaneous leprosy type 1 reactions. PloS Negl Trop D (2012) 6:e1869. doi: 10.1371/journal.pntd.0001869 PMC3486878 23133681

[64] Kurita M Araoka T Hishida T O’Keefe DD Takahashi Y Sakamoto A. *In vivo* reprogramming of wound-resident cells generates skin epithelial tissue. Nature (2018) 561:243–7. doi: 10.1038/s41586-018-0477-4 PMC9651909 30185909

[65] Amoh Y Li L Katsuoka K Hoffman RM. Multipotent hair follicle stem cells promote repair of spinal cord injury and recovery of walking function. Cell Cycle (2008) 7:1865–9. doi: 10.4161/cc.7.12.6056 18583926

[66] Mantovani A Sozzani S Locati M Allavena P Sica A. Macrophage polarization: tumor-associated macrophages as a paradigm for polarized M2 mononuclear phagocytes. Trends Immunol (2002) 23:549–55. doi: 10.1016/S1471-4906(02)02302-5 12401408

[67] Kibbie J Teles RM Wang Z Hong P Montoya D Krutzik S. Jagged1 instructs macrophage differentiation in leprosy. PloS Pathog (2016) 12:e1005808. doi: 10.1371/journal.ppat.1005808 27532668 PMC4988718

[68] Parker HA Forrester L Kaldor CD Dickerhof N Hampton MB. Antimicrobial activity of neutrophils against mycobacteria. Front Immunol (2021) 12:782495. doi: 10.3389/fimmu.2021.782495 35003097 PMC8732375

[69] Chattree V Khanna N Bisht V Rao DN. Inhibition of apoptosis, activation of NKT cell and upregulation of CD40 and CD40L mediated by M. leprae antigen(s) combined with Murabutide and Trat peptide in leprosy patients. Mol Cell Biochem (2008) 309:87–97. doi: 10.1007/s11010-007-9646-8 18008143

[70] Silva BJ Barbosa MG Andrade PR Ferreira H Nery JA Corte-Real S. Autophagy is an innate mechanism associated with leprosy polarization. PloS Pathog (2017) 13:e1006103. doi: 10.1371/journal.ppat.1006103 28056107 PMC5215777

[71] de Sousa JR Lucena NF Sotto MN Quaresma J. Immunohistochemical characterization of the M4 macrophage population in leprosy skin lesions. BMC Infect Dis (2018) 18:576. doi: 10.1186/s12879-018-3478-x 30442123 PMC6238386

[72] Quaresma TC de Aguiar VL de Sousa JR de Souza AT Fuzii HT Duarte M. Immunohistochemical characterization of M1, M2, and M4 macrophages in leprosy skin lesions. Pathogens (2023) 12(10):1225. doi: 10.3390/pathogens12101225 37887741 PMC10610015

[73] Oldenburg R Mayau V Prandi J Arbues A Astarie-Dequeker C Guilhot C. Mycobacterial phenolic glycolipids selectively disable TRIF-dependent TLR4 signaling in macrophages. Front Immunol (2018) 9:2. doi: 10.3389/fimmu.2018.00002 29403489 PMC5780341

[74] Ma Y Pei Q Zhang L Lu J Shui T Chen J. Live Mycobacterium leprae inhibits autophagy and apoptosis of infected macrophages and prevents engulfment of host cell by phagocytes. Am J Transl Res (2018) 10:2929–39. PMC6176229 30323879

[75] Yang D Chen J Shi C Jing Z Song N. Autophagy gene polymorphism is associated with susceptibility to leprosy by affecting inflammatory cytokines. Inflammation (2014) 37:593–8. doi: 10.1007/s10753-013-9773-1 24264476

[76] Schlesinger LS Horwitz MA. Phagocytosis of leprosy bacilli is mediated by complement receptors CR1 and CR3 on human monocytes and complement component C3 in serum. J Clin Invest (1990) 85:1304–14. doi: 10.1172/JCI114568 PMC296567 2138634

[77] Roy S Frodsham A Saha B Hazra SK Mascie-Taylor CG Hill AV. Association of vitamin D receptor genotype with leprosy type. J Infect Dis (1999) 179:187–91. doi: 10.1086/314536 9841838

[78] Grossi DOA Chaves AT Cardoso MS Pinheiro G Antunes DE Grossi M. Reduced vitamin D receptor (VDR) and cathelicidin antimicrobial peptide (CAMP) gene expression contribute to the maintenance of inflammatory immune response in leprosy patients. Microbes Infect (2022) 24:104981. doi: 10.1016/j.micinf.2022.104981 35462022

[79] Liu PT Wheelwright M Teles R Komisopoulou E Edfeldt K Ferguson B. MicroRNA-21 targets the vitamin D-dependent antimicrobial pathway in leprosy. Nat Med (2012) 18:267–73. doi: 10.1038/nm.2584 PMC3274599 22286305

[80] Sinsimer D Fallows D Peixoto B Krahenbuhl J Kaplan G Manca C. Mycobacterium leprae actively modulates the cytokine response in naive human monocytes. Infect Immun (2010) 78:293–300. doi: 10.1128/IAI.00816-09 19841079 PMC2798203

[81] de Sousa JR de Sousa R de Souza AT Dias LJ Oliveira CF Simoes QJ. Response of iNOS and its relationship with IL-22 and STAT3 in macrophage activity in the polar forms of leprosy. Acta Trop (2017) 171:74–9. doi: 10.1016/j.actatropica.2017.03.016 28327412

[82] Madigan CA Cambier CJ Kelly-Scumpia KM Scumpia PO Cheng TY Zailaa J. A macrophage response to mycobacterium leprae phenolic glycolipid initiates nerve damage in leprosy. Cell (2017) 170:973–85. doi: 10.1016/j.cell.2017.07.030 PMC5848073 28841420

[83] Realegeno S Kelly-Scumpia KM Dang AT Lu J Teles R Liu PT. S100A12 Is Part of the Antimicrobial Network against Mycobacterium leprae in Human Macrophages. PloS Pathog (2016) 12:e1005705. doi: 10.1371/journal.ppat.1005705 27355424 PMC4927120

[84] Wenk MR. Lipidomics of host-pathogen interactions. FEBS Lett (2006) 580:5541–51. doi: 10.1016/j.febslet.2006.07.007 16859687

[85] Tanigawa K Degang Y Kawashima A Akama T Yoshihara A Ishido Y. Essential role of hormone-sensitive lipase (HSL) in the maintenance of lipid storage in Mycobacterium leprae-infected macrophages. Microb Pathog (2012) 52:285–91. doi: 10.1016/j.micpath.2012.02.003 22553833

[86] Fukutomi Y Maeda Y Makino M. Apoptosis-inducing activity of clofazimine in macrophages. Antimicrob Agents CH (2011) 55:4000–5. doi: 10.1128/AAC.00434-11 PMC3165281 21690278

[87] Mattos KA Oliveira VC Berredo-Pinho M Amaral JJ Antunes LC Melo RC. Mycobacterium leprae intracellular survival relies on cholesterol accumulation in infected macrophages: a potential target for new drugs for leprosy treatment. Cell Microbiol (2014) 16:797–815. doi: 10.1111/cmi.12279 24552180 PMC4262048

[88] Rambukkana A Yamada H Zanazzi G Mathus T Salzer JL Yurchenco PD. Role of alpha-dystroglycan as a Schwann cell receptor for Mycobacterium leprae. Science (1998) 282:2076–9. doi: 10.1126/science.282.5396.2076 9851927

[89] Hedl M Abraham C. A TNFSF15 disease-risk polymorphism increases pattern-recognition receptor-induced signaling through caspase-8-induced IL-1. P Natl Acad Sci USA (2014) 111:13451–6. doi: 10.1073/pnas.1404178111 PMC4169936 25197060

[90] Borah K Girardi K Mendum TA Lery L Beste D Lara FA. Intracellular mycobacterium leprae utilizes host glucose as a carbon source in Schwann cells. MBIO (2019) 10:e02351-19. doi: 10.1128/mBio.02351-19 31848273 PMC6918074

[91] Masaki T Qu J Cholewa-Waclaw J Burr K Raaum R Rambukkana A. Reprogramming adult Schwann cells to stem cell-like cells by leprosy bacilli promotes dissemination of infection. Cell (2013) 152:51–67. doi: 10.1016/j.cell.2012.12.014 23332746 PMC4314110

[92] Diaz AC Dias AA Rosa T Batista-Silva LR Rosa PS Toledo-Pinto TG. PGL I expression in live bacteria allows activation of a CD206/PPARgamma cross-talk that may contribute to successful Mycobacterium leprae colonization of peripheral nerves. PloS Pathog (2018) 14:e1007151. doi: 10.1371/journal.ppat.1007151 29979790 PMC6056075

[93] Medeiros RC Girardi KD Cardoso FK Mietto BS Pinto TG Gomez LS. Subversion of Schwann cell glucose metabolism by mycobacterium leprae. J Biol Chem (2016) 291:21375–87. doi: 10.1074/jbc.M116.725283 PMC5076808 27555322

[94] Mabalay MC Helwig EB Tolentino JG Binford CH. The histopathology and histochemistry of erythema nodosum leprosum. Int J Lepr (1965) 33:28–49. 14282354

[95] Job CK Gude S Macaden VP. Erythema nodosum leprosum. a clinico-pathologic study. Int J Lepr (1964) 32:177–84. 14203324

[96] McAdam KP Anders RF Smith SR Russell DA Price MA. Association of amyloidosis with erythema nodosum leprosum reactions and recurrent neutrophil leucocytosis in leprosy. Lancet (1975) 2:572–3. doi: 10.1016/S0140-6736(75)90168-3 51405

[97] Schmitz V Prata RB Barbosa MG Mendes MA Brandao SS Amadeu TP. Expression of CD64 on circulating neutrophils favoring systemic inflammatory status in erythema nodosum leprosum. PloS Negl Trop D (2016) 10:e4955. doi: 10.1371/journal.pntd.0004955 PMC4996526 27556927

[98] Da SC Dias AA Da CNJ de Miranda MA Ferreira H Rodrigues TF. Neutrophil extracellular traps contribute to the pathogenesis of leprosy type 2 reactions. PloS Negl Trop D (2019) 13:e7368. doi: 10.1371/journal.pntd.0007368 PMC6736252 31504035

[99] Modlin RL Melancon-Kaplan J Young SM Pirmez C Kino H Convit J. Learning from lesions: patterns of tissue inflammation in leprosy. P Natl Acad Sci USA (1988) 85:1213–7. doi: 10.1073/pnas.85.4.1213 PMC279737 3257577

[100] Misra N Murtaza A Walker B Narayan NP Misra RS Ramesh V. Cytokine profile of circulating T cells of leprosy patients reflects both indiscriminate and polarized T-helper subsets: T-helper phenotype is stable and uninfluenced by related antigens of Mycobacterium leprae. Immunology (1995) 86:97–103. 7590888 PMC1383815

[101] de Sousa JR Sotto MN Simoes QJ. Leprosy as a complex infection: breakdown of the th1 and th2 immune paradigm in the immunopathogenesis of the disease. Front Immunol (2017) 8:1635. doi: 10.3389/fimmu.2017.01635 29234318 PMC5712391

[102] Schmitt E Klein M Bopp T. Th9 cells, new players in adaptive immunity. Trends Immunol (2014) 35:61–8. doi: 10.1016/j.it.2013.10.004 24215739

[103] Dersimonian H McAdam KP Mackworth-Young C Stollar BD. The recurrent expression of variable region segments in human IgM anti-DNA autoantibodies. J Immunol (1989) 142:4027–33. doi: 10.4049/jimmunol.142.11.4027 2497186

[104] Santos MB de Oliveira DT Cazzaniga RA Varjao CS Dos SP Santos M. Distinct roles of th17 and th1 cells in inflammatory responses associated with the presentation of paucibacillary leprosy and leprosy reactions. Scand J Immunol (2017) 86:40–9. doi: 10.1111/sji.12558 28426172

[105] de Lima SE de Sousa JR de Sousa AT Fuzii HT Dias JL Carneiro FR. New immunologic pathways in the pathogenesis of leprosy: role for Th22 cytokines in the polar forms of the disease. J Am Acad Dermatol (2015) 72:729–30. doi: 10.1016/j.jaad.2014.11.023 25773413

[106] Kumar S Naqvi RA Ali R Rani R Khanna N Rao DN. FoxP3 provides competitive fitness to CD4(+)CD25(+) T cells in leprosy patients via transcriptional regulation. Eur J Immunol (2014) 44:431–9. doi: 10.1002/eji.201343649 24214631

[107] Tarique M Saini C Naqvi RA Khanna N Rao DN. Increased IL-35 producing Tregs and CD19(+)IL-35(+) cells are associated with disease progression in leprosy patients. Cytokine (2017) 91:82–8. doi: 10.1016/j.cyto.2016.12.011 28038394

[108] Bobosha K Wilson L van Meijgaarden KE Bekele Y Zewdie M van der Ploeg-van SJ. T-cell regulation in lepromatous leprosy. PloS Negl Trop D (2014) 8:e2773. doi: 10.1371/journal.pntd.0002773 PMC3983090 24722473

[109] Saini C Siddiqui A Ramesh V Nath I. Leprosy reactions show increased th17 cell activity and reduced FOXP3+ Tregs with concomitant decrease in TGF-beta and increase in IL-6. PloS Negl Trop D (2016) 10:e4592. doi: 10.1371/journal.pntd.0004592 PMC4818038 27035913

[110] Agrawal S Parkash O Palaniappan AN Bhatia AK Kumar S Chauhan DS. Efficacy of T regulatory cells, th17 cells and the associated markers in monitoring tuberculosis treatment response. Front Immunol (2018) 9:157. doi: 10.3389/fimmu.2018.00157 29472922 PMC5810270

[111] Tarique M Naz H Kurra SV Saini C Naqvi RA Rai R. Interleukin-10 producing regulatory B cells transformed CD4(+)CD25(-) into tregs and enhanced regulatory T cells function in human leprosy. Front Immunol (2018) 9:1636. doi: 10.3389/fimmu.2018.01636 30083152 PMC6065098

[112] Saini C Tarique M Ramesh V Khanna N Sharma A. gammadelta T cells are associated with inflammation and immunopathogenesis of leprosy reactions. Immunol Lett (2018) 200:55–65. doi: 10.1016/j.imlet.2018.07.005 30006101

[113] Moraes MO Sampaio EP Nery JA Saraiva BC Alvarenga FB Sarno EN. Sequential erythema nodosum leprosum and reversal reaction with similar lesional cytokine mRNA patterns in a borderline leprosy patient. Brit J Dermatol (2001) 144:175–81. doi: 10.1046/j.1365-2133.2001.03970.x 11167702

[114] Verhagen C Faber W Klatser P Buffing A Naafs B Das P. Immunohistological analysis of in *situ* expression of mycobacterial antigens in skin lesions of leprosy patients across the histopathological spectrum. Association of Mycobacterial lipoarabinomannan (LAM) and Mycobacterium leprae phenolic glycolipid-I (PGL-I) with leprosy reactions. Am J Pathol (1999) 154:1793–804. doi: 10.1016/S0002-9440(10)65435-1 PMC3277205 10362804

[115] Mira MT Alcais A Van Thuc N Thai VH Huong NT Ba NN. Chromosome 6q25 is linked to susceptibility to leprosy in a Vietnamese population. Nat Genet (2003) 33:412–5. doi: 10.1038/ng1096 12577057

[116] Zhang FR Huang W Chen SM Sun LD Liu H Li Y. Genomewide association study of leprosy. N Engl J Med (2009) 361:2609–18. doi: 10.1056/NEJMoa0903753 20018961

[117] Zhang DF Wang D Li YY Yao YG. Mapping genetic variants in the CFH gene for association with leprosy in Han Chinese. Genes Immun (2014) 15:506–10. doi: 10.1038/gene.2014.43 25030427

[118] Liu H Bao F Irwanto A Fu X Lu N Yu G. An association study of TOLL and CARD with leprosy susceptibility in Chinese population. Hum Mol Genet (2013) 22:4430–7. doi: 10.1093/hmg/ddt286 PMC3792694 23784377

[119] Fava VM Dallmann-Sauer M Orlova M Correa-Macedo W Van Thuc N Thai VH. Deep resequencing identifies candidate functional genes in leprosy GWAS loci. PloS Negl Trop D (2021) 15:e10029. doi: 10.1371/journal.pntd.0010029 PMC8687567 34879060

[120] Liu H Wang Z Li Y Yu G Fu X Wang C. Genome-wide analysis of protein-coding variants in leprosy. J Invest Dermatol (2017) 137:2544–51. doi: 10.1016/j.jid.2017.08.004 28842327

[121] Zhang F Liu H Chen S Low H Sun L Cui Y. Identification of two new loci at IL23R and RAB32 that influence susceptibility to leprosy. Nat Genet (2011) 43:1247–51. doi: 10.1038/ng.973 22019778

[122] Leturiondo AL Noronha AB Mendonca C Ferreira CO Alvarado-Arnez LE Manta F. Association of NOD2 and IFNG single nucleotide polymorphisms with leprosy in the Amazon ethnic admixed population. PloS Negl Trop D (2020) 14:e8247. doi: 10.1371/journal.pntd.0008247 PMC7239438 32433683

[123] Ali S Srivastava AK Chopra R Aggarwal S Garg VK Bhattacharya SN. IL12B SNPs and copy number variation in IL23R gene associated with susceptibility to leprosy. J Med Genet (2013) 50:34–42. doi: 10.1136/jmedgenet-2012-101214 23240095

[124] Santos AR Suffys PN Vanderborght PR Moraes MO Vieira LM Cabello PH. Role of tumor necrosis factor-alpha and interleukin-10 promoter gene polymorphisms in leprosy. J Infect Dis (2002) 186:1687–91. doi: 10.1086/345366 12447749

[125] Moraes MO Pacheco AG Schonkeren JJ Vanderborght PR Nery JA Santos AR. Interleukin-10 promoter single-nucleotide polymorphisms as markers for disease susceptibility and disease severity in leprosy. Genes Immun (2004) 5:592–5. doi: 10.1038/sj.gene.6364122 15306847

[126] Malhotra D Darvishi K Sood S Sharma S Grover C Relhan V. IL-10 promoter single nucleotide polymorphisms are significantly associated with resistance to leprosy. Hum Genet (2005) 118:295–300. doi: 10.1007/s00439-005-0042-8 16163478

[127] Liu H Irwanto A Tian H Fu X Yu Y Yu G. Identification of IL18RAP/IL18R1 and IL12B as leprosy risk genes demonstrates shared pathogenesis between inflammation and infectious diseases. Am J Hum Genet (2012) 91:935–41. doi: 10.1016/j.ajhg.2012.09.010 PMC3487119 23103228

[128] Wong SH Gochhait S Malhotra D Pettersson FH Teo YY Khor CC. Leprosy and the adaptation of human toll-like receptor 1. PloS Pathog (2010) 6:e1000979. doi: 10.1371/journal.ppat.1000979 20617178 PMC2895660

[129] Marques CS Brito-de-Souza VN Guerreiro LT Martins JH Amaral EP Cardoso CC. Toll-like receptor 1 N248S single-nucleotide polymorphism is associated with leprosy risk and regulates immune activation during mycobacterial infection. J Infect Dis (2013) 208:120–9. doi: 10.1093/infdis/jit133 23547143

[130] Masin PS Visentin HA Elpidio L Sell AM Visentainer L Lima NQ. Genetic polymorphisms of toll-like receptors in leprosy patients from southern Brazil. Front Genet (2022) 13:952219. doi: 10.3389/fgene.2022.952219 36313452 PMC9596761

[131] Maciel-Fiuza MF Costa P Kowalski TW Schuler-Faccini L Bonamigo RR Vetoratto R. Evaluation of polymorphisms in toll-like receptor genes as biomarkers of the response to treatment of erythema nodosum leprosum. Front Med-Lausanne (2021) 8:713143. doi: 10.3389/fmed.2021.713143 35141236 PMC8819000

[132] Gutierrez-Castaneda LD Acosta CR Bustos MA Garcia DK Bohada DP Rodriguez R. Single nucleotide variants in the TLR1, TLR2 and TLR6 genes: A case-control study in a Colombian population. Trop Med Infect Dis (2023) 8:473. doi: 10.3390/tropicalmed8100473 PMC10610572 37888601

[133] Bochud PY Hawn TR Siddiqui MR Saunderson P Britton S Abraham I. Toll-like receptor 2 (TLR2) polymorphisms are associated with reversal reaction in leprosy. J Infect Dis (2008) 197:253–61. doi: 10.1086/524688 PMC3077295 18177245

[134] Long SY Wang L Jiang HQ Shi Y Zhang WY Xiong JS. Single-nucleotide polymorphisms related to leprosy risk and clinical phenotypes among Chinese population. Pharmacogen Pers Med (2021) 14:813–21. doi: 10.2147/PGPM.S314861 PMC8285297 34285550

[135] Gzara C Dallmann-Sauer M Orlova M Van Thuc N Thai VH Fava VM. Family-based genome-wide association study of leprosy in Vietnam. PloS Pathog (2020) 16:e1008565. doi: 10.1371/journal.ppat.1008565 32421744 PMC7259797

[136] Chopra R Kalaiarasan P Ali S Srivastava AK Aggarwal S Garg VK. PARK2 and proinflammatory/anti-inflammatory cytokine gene interactions contribute to the susceptibility to leprosy: a case-control study of North Indian population. BMJ Open (2014) 4:e4239. doi: 10.1136/bmjopen-2013-004239 PMC3939656 24578538

[137] Grant AV Alter A Huong NT Orlova M Van Thuc N Ba NN. Crohn’s disease susceptibility genes are associated with leprosy in the Vietnamese population. J Infect Dis (2012) 206:1763–7. doi: 10.1093/infdis/jis588 22984114

[138] Gilchrist JJ Auckland K Parks T Mentzer AJ Goldblatt L Naranbhai V. Genome-wide association study of leprosy in Malawi and Mali. PloS Pathog (2022) 18:e1010312. doi: 10.1371/journal.ppat.1010312 36121873 PMC9624411

[139] Covolo DSF Querino GA Mendes CR Nieto BDSV Bettoni BMP Tavora MM. HLA-DPB1 and HLA-C alleles are associated with leprosy in a Brazilian population. Hum Immunol (2021) 82:11–8. doi: 10.1016/j.humimm.2020.10.008 33189423

[140] Shankarkumar U Ghosh K Badakere S Mohanty D. Novel HLA class I alleles associated with Indian leprosy patients. J BioMed Biotechnol (2003) 2003:208–11. doi: 10.1155/S1110724303210019 PMC400212 12975536

[141] Alter A Huong NT Singh M Orlova M Van Thuc N Katoch K. Human leukocyte antigen class I region single-nucleotide polymorphisms are associated with leprosy susceptibility in Vietnam and India. J Infect Dis (2011) 203:1274–81. doi: 10.1093/infdis/jir024 PMC3069725 21459816

[142] Zhang F Liu H Chen S Wang C Zhu C Zhang L. Evidence for an association of HLA-DRB1*15 and DRB1*09 with leprosy and the impact of DRB1*09 on disease onset in a Chinese Han population. BMC Med Genet (2009) 10:133. doi: 10.1186/1471-2350-10-133 20003324 PMC2797507

[143] Vanderborght PR Pacheco AG Moraes ME Antoni G Romero M Verville A. HLA-DRB1*04 and DRB1*10 are associated with resistance and susceptibility, respectively, in Brazilian and Vietnamese leprosy patients. Genes Immun (2007) 8:320–4. doi: 10.1038/sj.gene.6364390 17396103

[144] Borras SG Cotorruelo C Racca L Recarte M Garcias C Biondi C. Association of leprosy with HLA-DRB1 in an Argentinean population. Ann Clin Biochem (2008) 45:96–8. doi: 10.1258/acb.2007.007156 18275683

[145] Alcais A Alter A Antoni G Orlova M Nguyen VT Singh M. Stepwise replication identifies a low-producing lymphotoxin-alpha allele as a major risk factor for early-onset leprosy. Nat Genet (2007) 39:517–22. doi: 10.1038/ng2000 17353895

[146] Cardoso CC Pereira AC Brito-de-Souza VN Duraes SM Ribeiro-Alves M Nery JA. TNF -308G>A single nucleotide polymorphism is associated with leprosy among Brazilians: a genetic epidemiology assessment, meta-analysis, and functional study. J Infect Dis (2011) 204:1256–63. doi: 10.1093/infdis/jir521 21917899

[147] Fava VM Cobat A Van Thuc N Latini AC Stefani MM Belone AF. Association of TNFSF8 regulatory variants with excessive inflammatory responses but not leprosy per se. J Infect Dis (2015) 211:968–77. doi: 10.1093/infdis/jiu566 25320285

[148] Zhang DF Huang XQ Wang D Li YY Yao YG. Genetic variants of complement genes ficolin-2, mannose-binding lectin and complement factor H are associated with leprosy in Han Chinese from Southwest China. Hum Genet (2013) 132:629–40. doi: 10.1007/s00439-013-1273-8 23423485

[149] Boldt A Oliveira-Tore CF Kretzschmar GC Weinschutz MH Stinghen ST Andrade FA. Hepatitis B virus infection among leprosy patients: A case for polymorphisms compromising activation of the lectin pathway and complement receptors. Front Immunol (2020) 11:574457. doi: 10.3389/fimmu.2020.574457 33643280 PMC7904891

[150] de Messias-Reason I Kremsner PG Kun JF. Functional haplotypes that produce normal ficolin-2 levels protect against clinical leprosy. J Infect Dis (2009) 199:801–4. doi: 10.1086/597070 19434912

[151] Mira MT Alcais A Nguyen VT Moraes MO Di Flumeri C Vu HT. Susceptibility to leprosy is associated with PARK2 and PACRG. Nature (2004) 427:636–40. doi: 10.1038/nature02326 14737177

[152] Chopra R Ali S Srivastava AK Aggarwal S Kumar B Manvati S. Mapping of PARK2 and PACRG overlapping regulatory region reveals LD structure and functional variants in association with leprosy in unrelated Indian population groups. PloS Genet (2013) 9:e1003578. doi: 10.1371/journal.pgen.1003578 23861666 PMC3701713

[153] Wang D Feng JQ Li YY Zhang DF Li XA Li QW. Genetic variants of the MRC1 gene and the IFNG gene are associated with leprosy in Han Chinese from Southwest China. Hum Genet (2012) 131:1251–60. doi: 10.1007/s00439-012-1153-7 22392581

[154] Alter A de Leseleuc L Van Thuc N Thai VH Huong NT Ba NN. Genetic and functional analysis of common MRC1 exon 7 polymorphisms in leprosy susceptibility. Hum Genet (2010) 127:337–48. doi: 10.1007/s00439-009-0775-x PMC2891106 20035344

[155] Tiyo BT Vendramini E de Souza VH Colli CM Alves HV Sell AM. Association of MBL2 exon 1 polymorphisms with multibacillary leprosy. Front Immunol (2020) 11:1927. doi: 10.3389/fimmu.2020.01927 33013845 PMC7494844

[156] Vasconcelos LR Fonseca JP Do CR de Mendonca TF Pereira VR Lucena-Silva N. Mannose-binding lectin serum levels in patients with leprosy are influenced by age and MBL2 genotypes. Int J Infect Dis (2011) 15:e551–7. doi: 10.1016/j.ijid.2011.04.008 21640628

[157] Cardona-Pemberthy V Rendon M Beltran JC Soto-Ospina A Munoz-Gomez A Araque-Marin P. Genetic variants, structural, and functional changes of Myelin Protein Zero and Mannose-Binding Lectin 2 protein involved in immune response and its allelic transmission in families of patients with leprosy in Colombia. Infect Genet Evol (2018) 61:215–23. doi: 10.1016/j.meegid.2018.04.002 29627640

[158] Marcinek P Jha AN Shinde V Sundaramoorthy A Rajkumar R Suryadevara NC. LRRK2 and RIPK2 variants in the NOD 2-mediated signaling pathway are associated with susceptibility to Mycobacterium leprae in Indian populations. PloS One (2013) 8:e73103. doi: 10.1371/journal.pone.0073103 24015287 PMC3756038

[159] Wang D Xu L Lv L Su LY Fan Y Zhang DF. Association of the LRRK2 genetic polymorphisms with leprosy in Han Chinese from Southwest China. Genes Immun (2015) 16:112–9. doi: 10.1038/gene.2014.72 25521227

[160] Paz J Silvestre M Moura LS Furlaneto IP Rodrigues YC Lima K. Association of the polymorphism of the vitamin D receptor gene (VDR) with the risk of leprosy in the Brazilian Amazon. Bioscience Rep (2021) 41:BSR20204102. doi: 10.1042/BSR20204102 PMC8264180 34143211

[161] Singh I Lavania M Pathak VK Ahuja M Turankar RP Singh V. VDR polymorphism, gene expression and vitamin D levels in leprosy patients from North Indian population. PloS Negl Trop D (2018) 12:e6823. doi: 10.1371/journal.pntd.0006823 PMC6286024 30481178

[162] Cardoso CC Pereira AC Brito-de-Souza VN Dias-Baptista IM Maniero VC Venturini J. IFNG +874 T>A single nucleotide polymorphism is associated with leprosy among Brazilians. Hum Genet (2010) 128:481–90. doi: 10.1007/s00439-010-0872-x 20714752

[163] Liu H Irwanto A Fu X Yu G Yu Y Sun Y. Discovery of six new susceptibility loci and analysis of pleiotropic effects in leprosy. Nat Genet (2015) 47:267–71. doi: 10.1038/ng.3212 25642632

[164] Sales-Marques C Salomao H Fava VM Alvarado-Arnez LE Amaral EP Cardoso CC. NOD2 and CCDC122-LACC1 genes are associated with leprosy susceptibility in Brazilians. Hum Genet (2014) 133:1525–32. doi: 10.1007/s00439-014-1502-9 25367361

[165] Wang D Fan Y Malhi M Bi R Wu Y Xu M. Missense variants in HIF1A and LACC1 contribute to leprosy risk in Han Chinese. Am J Hum Genet (2018) 102:794–805. doi: 10.1016/j.ajhg.2018.03.006 29706348 PMC5986702

[166] Schenk M Krutzik SR Sieling PA Lee DJ Teles RM Ochoa MT. NOD2 triggers an interleukin-32-dependent human dendritic cell program in leprosy. Nat Med (2012) 18:555–63. doi: 10.1038/nm.2650 PMC3348859 22447076

[167] Jarduli LR Alves HV de Souza-Santana FC Marcos EV Pereira AC Dias-Baptista IM. Influence of KIR genes and their HLA ligands in the pathogenesis of leprosy in a hyperendemic population of Rondonopolis, Southern Brazil. BMC Infect Dis (2014) 14:438. doi: 10.1186/1471-2334-14-438 25117794 PMC4141108

[168] Franceschi DS Mazini PS Rudnick CC Sell AM Tsuneto LT de Melo FC. Association between killer-cell immunoglobulin-like receptor genotypes and leprosy in Brazil. Tissue Antigens (2008) 72:478–82. doi: 10.1111/j.1399-0039.2008.01127.x 18778326

[169] Shi W Mi Z Wang Z Zhang H Wang N Wang Z. Massively parallel sequencingof the filaggrin gene reveals an association between FLG loss-of-function mutations and leprosy. Acta Derm-Venereol (2020) 100:v299. doi: 10.2340/00015555-3663 PMC9274930 33047146

[170] Schuring RP Hamann L Faber WR Pahan D Richardus JH Schumann RR. Polymorphism N248S in the human Toll-like receptor 1 gene is related to leprosy and leprosy reactions. J Infect Dis (2009) 199:1816–9. doi: 10.1086/599121 19456232

[171] Dallmann-Sauer M Xu YZ Da CA Tao S Gomes TA Prata R. Allele-dependent interaction of LRRK2 and NOD2 in leprosy. PloS Pathog (2023) 19:e1011260. doi: 10.1371/journal.ppat.1011260 36972292 PMC10079233

[172] Hill AV. Aspects of genetic susceptibility to human infectious diseases. Annu Rev Genet (2006) 40:469–86. doi: 10.1146/annurev.genet.40.110405.090546 17094741

[173] Aguilar-Medina M Escamilla-Tilch M Frias-Castro LO Romero-Quintana G Estrada-Garcia I Estrada-Parra S. HLA alleles are genetic markers for susceptibility and resistance towards leprosy in a Mexican Mestizo population. Ann Hum Genet (2017) 81:35–40. doi: 10.1111/ahg.12183 28025823

[174] de Souza-Santana FC Marcos EV Nogueira ME Ura S Tomimori J. Human leukocyte antigen class I and class II alleles are associated with susceptibility and resistance in borderline leprosy patients from Southeast Brazil. BMC Infect Dis (2015) 15:22. doi: 10.1186/s12879-015-0751-0 25605482 PMC4307149

[175] Dallmann-Sauer M Fava VM Gzara C Orlova M Van Thuc N Thai VH. The complex pattern of genetic associations of leprosy with HLA class I and class II alleles can be reduced to four amino acid positions. PloS Pathog (2020) 16:e1008818. doi: 10.1371/journal.ppat.1008818 32776973 PMC7440659

[176] Zhang X Cheng Y Zhang Q Wang X Lin Y Yang C. Meta-analysis identifies major histocompatiblity complex loci in or near HLA-DRB1, HLA-DQA1, HLA-C as associated with leprosy in Chinese Han population. J Invest Dermatol (2019) 139:957–60. doi: 10.1016/j.jid.2018.09.029 30389493

[177] Shinde V Marcinek P Rani DS Sunder SR Arun S Jain S. Genetic evidence of TAP1 gene variant as a susceptibility factor in Indian leprosy patients. Hum Immunol (2013) 74:803–7. doi: 10.1016/j.humimm.2013.01.001 23395648

[178] Da SC Sampaio L Costa MB de Paula CCM Sadissou IA de Oliveira RCM. Analysis of HLA-G protein expression in leprosy. Immunogenetics (2020) 72:333–7. doi: 10.1007/s00251-020-01168-4 32556498

[179] Tosh K Ravikumar M Bell JT Meisner S Hill AV Pitchappan R. Variation in MICA and MICB genes and enhanced susceptibility to paucibacillary leprosy in South India. Hum Mol Genet (2006) 15:2880–7. doi: 10.1093/hmg/ddl229 16923796

[180] Fava VM Xu YZ Lettre G Van Thuc N Orlova M Thai VH. Pleiotropic effects for Parkin and LRRK2 in leprosy type-1 reactions and Parkinson’s disease. P Natl Acad Sci USA (2019) 116:15616–24. doi: 10.1073/pnas.1901805116 PMC6681704 31308240

[181] Zhang L Wang K Lei Y Li Q Nice EC Huang C. Redox signaling: Potential arbitrator of autophagy and apoptosis in therapeutic response. Free Radical Bio Med (2015) 89:452–65. doi: 10.1016/j.freeradbiomed.2015.08.030 26454086

[182] Yang Y Jiang G Zhang P Fan J. Programmed cell death and its role in inflammation. Military Med Res (2015) 2:1–12. doi: 10.1186/s40779-015-0039-0 PMC4455968 26045969

[183] Pepineli AC Alves HV Tiyo BT Macedo LC Visentainer L de Lima NQ. Vitamin D receptor gene polymorphisms are associated with leprosy in Southern Brazil. Front Immunol (2019) 10:2157. doi: 10.3389/fimmu.2019.02157 31636627 PMC6787522

[184] Alves HV de Moraes AG Pepineli AC Tiyo BT de Lima NQ Santos T. The impact of KIR/HLA genes on the risk of developing multibacillary leprosy. PloS Negl Trop D (2019) 13:e7696. doi: 10.1371/journal.pntd.0007696 PMC6762192 31525196

[185] Kretzschmar GC Oliveira LC Nisihara RM Velavan TP Stinghen ST Stahlke E. Complement receptor 1 (CR1, CD35) association with susceptibility to leprosy. PloS Negl Trop D (2018) 12:e6705. doi: 10.1371/journal.pntd.0006705 PMC6103516 30092084

[186] Krarup A Thiel S Hansen A Fujita T Jensenius JC. L-ficolin is a pattern recognition molecule specific for acetyl groups. J Biol Chem (2004) 279:47513–9. doi: 10.1074/jbc.M407161200 15331601

[187] Dornelles LN Pereira-Ferrari L Messias-Reason I. Mannan-binding lectin plasma levels in leprosy: deficiency confers protection against the lepromatous but not the tuberculoid forms. Clin Exp Immunol (2006) 145:463–8. doi: 10.1111/j.1365-2249.2006.03161.x PMC1809702 16907914

[188] Aggarwal S Ali S Chopra R Srivastava A Kalaiarasan P Malhotra D. Genetic variations and interactions in anti-inflammatory cytokine pathway genes in the outcome of leprosy: a study conducted on a MassARRAY platform. J Infect Dis (2011) 204:1264–73. doi: 10.1093/infdis/jir516 21917900

[189] Ali S Chopra R Aggarwal S Srivastava AK Kalaiarasan P Malhotra D. Association of variants in BAT1-LTA-TNF-BTNL2 genes within 6p21.3 region show graded risk to leprosy in unrelated cohorts of Indian population. Hum Genet (2012) 131:703–16. doi: 10.1007/s00439-011-1114-6 22071774

[190] Teles R Kelly-Scumpia KM Sarno EN Rea TH Ochoa MT Cheng G. IL-27 suppresses antimicrobial activity in human leprosy. J Invest Dermatol (2015) 135:2410–7. doi: 10.1038/jid.2015.195 PMC4567935 26030183

[191] Fava VM Sales-Marques C Alcais A Moraes MO Schurr E. Age-dependent association of TNFSF15/TNFSF8 variants and leprosy type 1 reaction. Front Immunol (2017) 8:155. doi: 10.3389/fimmu.2017.00155 28261213 PMC5306391

[192] Wang Z Sun Y Fu X Yu G Wang C Bao F. A large-scale genome-wide association and meta-analysis identified four novel susceptibility loci for leprosy. Nat Commun (2016) 7:13760. doi: 10.1038/ncomms13760 27976721 PMC5172377

[193] Soares CT Fachin L Trombone A Rosa PS Ghidella CC Belone A. Potential of AKR1B10 as a biomarker and therapeutic target in type 2 leprosy reaction. Front Med-Lausanne (2018) 5:263. doi: 10.3389/fmed.2018.00263 30320113 PMC6166685

[194] Khadge S Banu S Bobosha K van der Ploeg-van SJ Goulart IM Thapa P. Longitudinal immune profiles in type 1 leprosy reactions in Bangladesh, Brazil, Ethiopia and Nepal. BMC Infect Dis (2015) 15:477. doi: 10.1186/s12879-015-1128-0 26510990 PMC4625471

[195] BroChado MJ Gatti MF Zago MA Roselino AM. Association of the solute carrier family 11 member 1 gene polymorphisms with susceptibility to leprosy in a Brazilian sample. Mem I Oswaldo Cruz (2016) 111:101–5. doi: 10.1590/0074-02760150326 PMC4750449 26814595

[196] Roger M Levee G Chanteau S Gicquel B Schurr E. No evidence for linkage between leprosy susceptibility and the human natural resistance-associated macrophage protein 1 (NRAMP1) gene in French Polynesia. Int J Lepr Other Mycobact Dis (1997) 65:197–202. 9251591

[197] Belone AF Rosa PS Trombone AP Fachin LR Guidella CC Ura S. Genome-wide screening of mRNA expression in leprosy patients. Front Genet (2015) 6:334. doi: 10.3389/fgene.2015.00334 26635870 PMC4653304

[198] Soares CT Trombone A Fachin L Rosa PS Ghidella CC Ramalho RF. Differential expression of microRNAs in leprosy skin lesions. Front Immunol (2017) 8:1035. doi: 10.3389/fimmu.2017.01035 28970833 PMC5609578

[199] Park AJ Rendini T Martiniuk F Levis WR. Leprosy as a model to understand cancer immunosurveillance and T cell anergy. J Leukocyte Biol (2016) 100:47–54. doi: 10.1189/jlb.5RU1215-537RR 27106673

[200] Ramos GB Salomao H Francio AS Fava VM Werneck RI Mira MT. Association analysis suggests SOD2 as a newly identified candidate gene associated with leprosy susceptibility. J Infect Dis (2016) 214:475–8. doi: 10.1093/infdis/jiw170 27132285

[201] Wang D Zhang DF Li GD Bi R Fan Y Wu Y. A pleiotropic effect of the APOE gene: association of APOE polymorphisms with multibacillary leprosy in Han Chinese from Southwest China. Brit J Dermatol (2018) 178:931–9. doi: 10.1111/bjd.16020 28977675

[202] Hottat F Coene M Cocito C. DNA methylation in leprosy-associated bacteria: Mycobacterium leprae and Corynebacterium tuberculostearicum. Med Microbiol Immun (1988) 177:33–45. doi: 10.1007/BF00190309 2828900

